# Nebulized corticosteroids *versus* systemic corticosteroids for patients with acute exacerbation of chronic obstructive pulmonary disease: A systematic review and meta-analysis comparing the benefits and harms reported by observational studies and randomized controlled trials

**DOI:** 10.3389/fphar.2022.966637

**Published:** 2022-10-05

**Authors:** Han-Shuo Hu, Zhuo Wang, Li-Mei Zhao, Xiao-Dong Liu

**Affiliations:** ^1^ Department of Pharmacy, Shengjing Hospital of China Medical University, Shenyang, China; ^2^ Department of The Second Clinical Pharmacy, School of Pharmacy, China Medical University, Shenyang, China

**Keywords:** acute exacerbation of chronic obstructive pulmonary disease, nebulized corticosteroids, observational studies, randomized controlled trial, real-world study, systemic corticosteroids

## Abstract

**Objective:** Systematic comparison of the efficacy and safety of nebulized corticosteroids and systemic corticosteroids for treating acute exacerbation of chronic obstructive pulmonary disease reported by high-quality, real-world observational studies and randomized controlled trials.

**Methods:** MEDLINE, EMBASE, and Cochrane Library databases were searched from the database creation date to 1 April 2022. Eligible observational studies and randomized controlled trials with changes in lung function and blood gas analysis results as the primary endpoints of interest, and the numbers of deteriorations and adverse events as the secondary endpoints were sought.

**Results:** Of the 2,837 identified studies, 22 were eligible and included in our analysis (N = 5,764 patients). Compared with systemic corticosteroids, nebulized corticosteroids resulted in comparable improvements in predicted FEV_1_%, FEV_1_, PaO_2_, PaCO_2_, and SaO_2_ at the treatment endpoint; however, observational studies reported more significant treatment outcomes with nebulized corticosteroids for FEV_1_ [mean difference, 0.26; 95% confidence interval (CI), 0.17–0.35; *p* < 0.005]. In terms of adverse reactions, the risks of gastrointestinal symptoms were 11% [Log risk ratio (LogRR) = 0.10; 95% confidence interval, 0.05–0.15; *p* < 0.005] higher for systemic corticosteroids than for nebulized corticosteroids in randomized controlled trials, while the risks of hyperglycemia were 6% (LogRR = 0.06; 95% CI, 0.01–0.11; *p* = 0.01) and 13% (LogRR = 0.12; 95% CI, 0.09–0.16; *p* < 0.005) higher in observational studies and randomized controlled trials, respectively.

**Conclusion:** According to our meta-analysis, either study type supported that nebulized corticosteroids can be used as an alternative to systemic corticosteroids for treating acute exacerbation of the chronic obstructive pulmonary disease. However, more well-designed prospective studies are needed to determine the optimal dose of nebulized corticosteroids and the advantages of sequential therapy.

## 1 Introduction

Chronic obstructive pulmonary disease (COPD) is a preventable and chronic inflammatory disease resulting in persistent airflow limitation that can cause extrapulmonary adverse effects. It has been identified as a disease of concern by the World Health Organization’s Chronic Disease Mortality Reduction Initiative 2030 (Collaborators, 2020). Currently, it has the third highest global mortality rate. Because of population growth and aging, COPD is expected to be the fourth leading cause of loss of life years by 2040 (Viegi et al., 2020). Acute exacerbation of COPD (AECOPD) refers to the exacerbation of respiratory symptoms such as cough and sputum in patients and the need to change medication regimens. The frequency and severity of acute exacerbations are directly related to patient mortality, negatively impact lung function and healing, and constantly increase the economic and social burden (Baqdunes et al., 2021; Chen and Chen, 2021; Macleod et al., 2021); therefore, preventing deterioration or recurrence and rapidly restoring health levels are key to the treatment of COPD.

The application of corticosteroids is an important clinical initiative for the treatment of AECOPD (Walters et al., 2018), and the administration of both nebulized corticosteroids (NCs) and systemic corticosteroids (SCs), including oral and intravenous routes, is recommended by the Global Initiative for Chronic Obstructive Pulmonary Disease guidelines for AECOPD. Acute exacerbation is a change in the bronchial state of the lungs caused by immune and cytokine stimulation by the organism in response to a causative agent. However, whether this state is caused by a systemic inflammatory response or a local inflammatory response in the lungs is unknown. This discussion has been ongoing, and the two forms of therapeutics associated with AECOPD, NCs, and SCs, have been the focus of comparisons. The Global Initiative for Chronic Obstructive Pulmonary Disease guidelines 2022 state that the benefits of using SCs are shorter recovery time, improved lung function and arterial hypoxemia, and a reduced risk of early relapse.

Corticosteroid-related complications include oral fungal infections, gastrointestinal bleeding, blood glucose fluctuation, decreased immunity, osteoporosis, and sepsis (Ernst et al., 2017; Caramori et al., 2019; Mkorombindo and Dransfield, 2020; Grosso et al., 2021), are extremely harmful. Therefore, clinicians have been searching for drugs that achieve the same therapeutic outcomes as SCs for AECOPD but produce fewer adverse effects. According to the current literature, studies have reported the comparable efficacy of nebulized inhaled budesonide (6 mg/d) and intravenous methylprednisolone (40 mg/d) for the treatment of AECOPD (Ding et al., 2016; Qi et al., 2021). According to a large Cochrane review, there was no difference in treatment failure, relapse, or mortality frequency among those receiving non-gut and oral corticosteroids (Walters et al., 2014). Currently, oral corticosteroids are as effective as intravenous injections for patients with worsening COPD. Compared with high-dose intravenous injections, lower doses of oral glucocorticosteroids do not lead to worse outcomes (de Jong et al., 2007; Lindenauer et al., 2010). At the same time, NCs have a relatively faster onset of action and fewer adverse effects than SCs, so they have become increasingly popular in recent years. The application of corticosteroid for AECOPD to achieve optimal clinical outcomes remains controversial. However, the real-world evidence summary literature for NCs *versus* SCs is virtually nonexistent. Real-world data during acute exacerbations cannot be ignored, and even data that cannot be simulated with certain randomized controlled trial would be better validated.

Observational studies have received increasing attention in recent years. Laboratory indicators for clinical patients in such studies are not subject to placebo or anti-placebo effects and are based on different populations worldwide for comparisons, thus reducing the possibility of false-positive or false-negative results; however, their heterogeneity is high, and it is crucial to control and address the heterogeneity using statistical methods (Marchenko et al., 2018). This study included randomized controlled trials and observational studies. The results of these two types of literature were compared to investigate whether the effects of setting-specific and real-world NCs and SCs for AECOPD treatment are consistent, to compare the presence of inconsistent and meaningful outcome indicators, and to compare efficacy outcomes. Attention was also focused on which corticosteroids are more appropriate as the choice at different doses to maximize the clinical patient benefits. Our study would fill the gap in the aggregation of real-world evidence for NCs *versus* SCs while comparing whether the results from the two types of research methods were consistent.

## 2 Methods

This study was a systematic review and meta-analysis performed according to the Preferred Reporting Items for Systematic Evaluations and Meta-Analysis (PRISMA) 2020 list, and it was designed to meet the requirements of the PRISMA guidelines (Page et al., 2021). The protocol of this systematic evaluation and meta-analysis is registered in the international Prospective Registry of Systematic Evaluation (PROSPERO) database (identifier number CRD42022321705, https://www.crd.york.ac.uk/prospero/display_record.php?ID=CRD42022321705).

### 2.1 Data sources and searches

We conducted an extensive search of EMBASE, Medline, The Cochrane Library databases, ClinicalTrials.gov, and Google Scholar (from build to 1 April 2022). The search strategy included the following keywords: “pulmonary disease, chronic obstructive,” “nebulized corticosteroid,” and “systemic corticosteroid” (Supplementary File S1). Additionally, the reference list of relevant articles was manually searched for other studies that might be eligible. If the reported data were unclear, we contacted the original authors.

### 2.2 Study selection and outcomes

Eligible studies were selected based on the inclusion and exclusion criteria. The inclusion criteria were as follows: randomized controlled trials or observational studies (e.g., cohort and case-control studies) of the efficacy of NCs compared with SCs for the treatment of AECOPD; patients diagnosed with AECOPD who met Global Initiative for Chronic Obstructive Lung Disease or ATS criteria; reported outcomes including pulmonary function reports, blood gas analysis results, worsening, or adverse events; and literature searches were not limited by the language of the published article or abstract. Exclusion criteria were as follows: conference abstracts, letters, case reports, review articles, preclinical studies, other nonrelevant studies and studies that did not report one of the outcomes of interest, and trials with less than fifteen patients in one group.

We attempted to obtain the adjusted or matched data for observational studies to minimize confounding. If multiple observational studies from the same data source were identified, then the literature with the longest study period reporting adjusted data was included. Two authors (H-SH and ZW) independently reviewed each title and abstract and assessed the full text of the retrieved studies; they attempted to resolve disagreements, if any, through consultation with the corresponding author.

### 2.3 Data extraction and quality assessment

Two reviewers (H-SH and ZW) independently extracted the following relevant information from each eligible study using a pre-designed form: study characteristics (authors’ names, year of publication, type of study design, treatment duration, treatment comparisons, additional treatments); patient characteristics (age, sex, current status); outcomes of interest (predicted FEV_1_% [FEV_1_%pred]; FEV_1_; changes in PaO_2_, PaCO_2_, and SaO_2_; treatment efficiency; adverse events such as hyperglycemia). Disagreements that existed were discussed first. If necessary, the corresponding author (X-DL) performed assistance and reached a consensus with all investigators. We collected and supplemented data from the previous meta-analyses for included studies with missing baseline standard deviation (SD) changes. When a study had multiple arms with different doses (e.g., budesonide 3 mg/d and 6 mg/d), we combined the results of the arms with different doses and calculated them as a group.

We assessed the methodological quality of each included randomized controlled trial using the Cochrane Risk of Bias Tool (Higgins et al., 2011). The assessment criteria were derived from selection bias, performance bias, detection bias, attrition bias, reporting bias, and other bias. Because observational studies had a higher risk of bias than randomized controlled trials, they were assessed using the Newcastle-Ottawa Quality Assessment Scale (Stang, 2010) and categorized as low-risk, moderate-risk, or high-risk based on item scores. The quality of each study was assessed by one reviewer and validated by another (H-SH and ZW); disagreements were resolved by consensus.

### 2.4 Data synthesis and statistical analysis

To compare the advantages and disadvantages of NCs and SCs, this meta-analysis with a random-effects model was performed to pool the data. If an outcome indicator was reported in multiple works, then the best data were selected based on the quality of the literature, time of publication, and number of patients. Data for dichotomous variables of outcomes, such as adverse events, were presented as effect measures with the adjusted Log risk ratio (LogRR) and 95% confidence interval (CI), which ensured normal distribution of the data. The effect size could be attributed to any value centered at 0 (0 means no effect). Data for continuous variables, such as lung function or blood gas analysis results, were reported as effect measures with mean difference (MD). I^2^ values were used to represent heterogeneity, with 25%, 50%, and 75% indicating low, medium, and high heterogeneity, respectively.

Additionally, if there was medium or high heterogeneity (I^2^ > 50%) in the results of a particular group, then a subgroup analysis of included studies was performed according to age (older than 40 years, older than 50 years, older than 60 years, and older than 70 years), drug doses and types (NCs, SCs), follow-up time (5, 7, and 10 days), number of patients, and sex ratio (Supplementary File S1). The doses of different corticosteroid drugs have been uniformly converted. To test the robustness of the results, the reviewers performed a sensitivity analysis with a sequential exclusion for each study in the pool. A quantitative analysis was performed using funnel plots (when more than 10 studies were included), Begg’s test, and Egger’s test to evaluate publication bias (Page et al., 2021). A meta-analysis was conducted using Stata version 17 software (Stata Corp, College Station, TX, United States), with a *p*-value < 0.05 considered statistically significant.

### 2.5 Ethical issues

This retrospective meta-analysis examined the literature and did not involve direct contact with patients. Therefore, ethics committee approval was not required for our study.

## 3 Results

**FIGURE 1:**
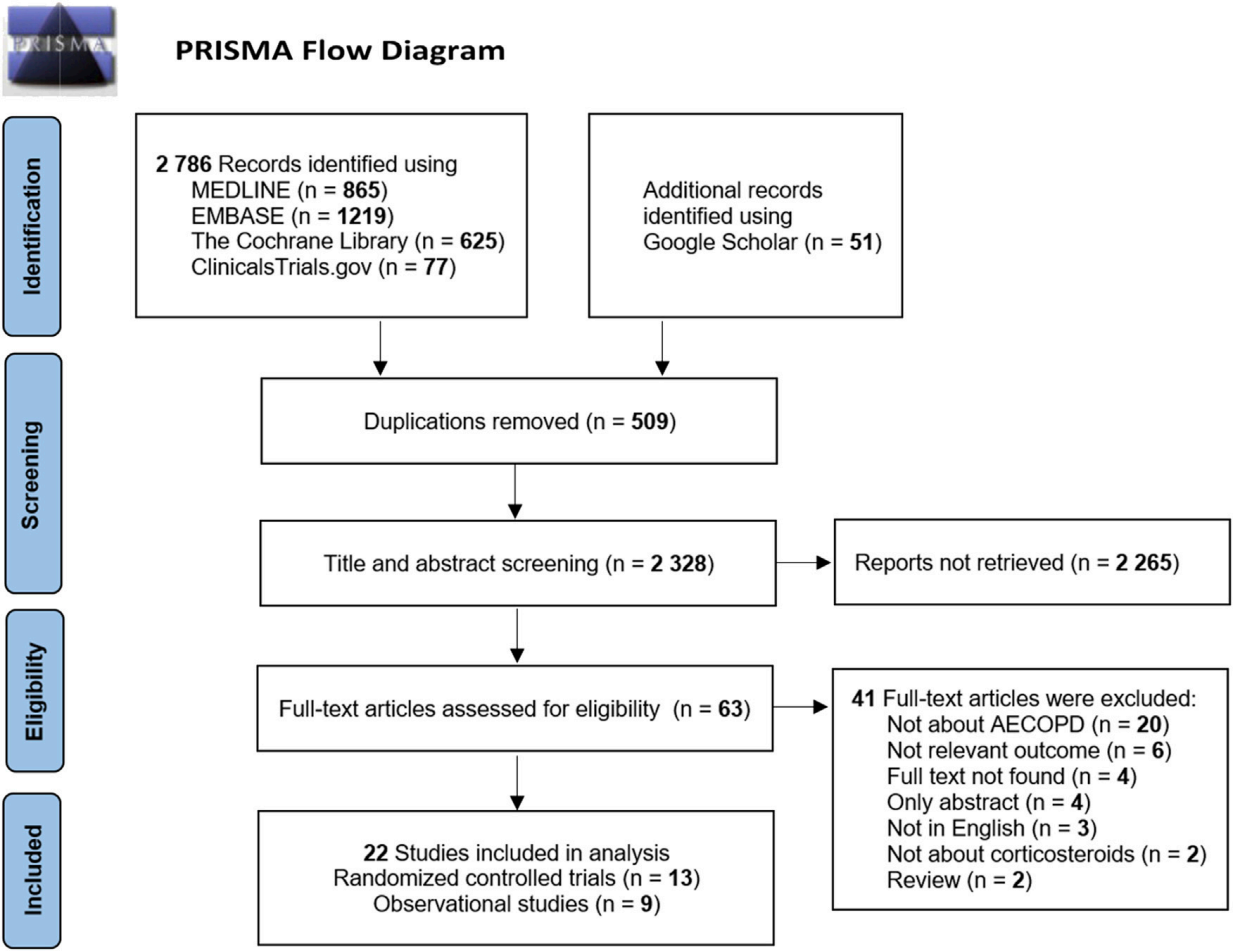
Flow chart of the literature screening process.

A total of 2,786 cited studies were identified in the initial search from the database and were supplemented by the inclusion of 51 additional articles from Google Scholar. The literature screening was performed for these 2,837 publications. After removing duplicates, 2,328 studies were identified. The initial screening of titles and abstracts yielded 63 articles for full-text reading according to the drug and population evaluation in the study. Finally, a total of 13 randomized controlled trials (Maltais et al., 2002; Mirici et al., 2003; Zhou and Han, 2004; Gunen et al., 2007; Zhang et al., 2011; Gong et al., 2013; Nemagouda, 2014; Sun et al., 2014; Yilmazel Ucar et al., 2014; Ding et al., 2016; Kafee et al., 2016; Zhao et al., 2019; Xiao et al., 2020) and nine observational studies (Wang and Chen, 2014; Song and Hu, 2015; Zhao, 2016; Shi and Geng, 2018; Zheng et al., 2019; Chen et al., 2020; Jiang, 2020; Gu, 2021; Liao et al., 2021) were included after the layer-by-layer screening. The literature screening process and results are shown in Figure 1.

### 3.1 Characteristics of included studies

The authors and year of publication of the literature, the number of cases in each study group, male-to-female ratio, age, interventions, and Newcastle-Ottawa Scale scores of the observational studies are presented in Supplementary File S1. In the included studies, 5,764 patients with AECOPD (all older than 40 years) were recruited. Only three studies (Nemagouda, 2014; Chen et al., 2020; Jiang, 2020) did not mention whether corticosteroids were used before patient admission, whereas the other studies used different time durations of having received corticosteroids treatment (last 24 h, 1 month, 3 months, or long-term) as exclusion criteria. Most study participants were current or former smokers with moderate or severe COPD. The included studies reported outcomes from 5 to 12 days post-treatment and used different doses of NCs and SCs as experimental *versus* control groups; however, they were much higher than the doses used for stable patients. Our current meta-analysis focused on changes in lung function (FEV_1_%pred, FEV_1_), arterial blood gas test results (PaO_2_, PaCO_2_, and SaO_2_), treatment efficiency, and outcomes of adverse events, including exacerbations.

In the 13 randomized controlled trials that met the inclusion criteria for efficacy assessment, the drug for the experimental group was nebulized inhaled budesonide; five studies (Mirici et al., 2003; Gunen et al., 2007; Gong et al., 2013; Yilmazel Ucar et al., 2014; Ding et al., 2016) used intravenous administration as control group, three studies (Maltais et al., 2002; Zhou and Han, 2004; Kafee et al., 2016) used oral administration, and five studies (Zhou and Han, 2004; Zhang et al., 2011; Nemagouda, 2014; Sun et al., 2014; Xiao et al., 2020) used both modes of administration. Nine observational studies also used nebulized inhaled budesonide for the experimental group; intravenous administration was used in seven of those studies (Wang and Chen, 2014; Song and Hu, 2015; Zhao, 2016; Shi and Geng, 2018; Jiang, 2020; Gu, 2021; Liao et al., 2021), and two studies (Zheng et al., 2019; Chen et al., 2020) used both oral and intravenous methods of administration. The mean age of all patients was 40 and 80 years. Patients in observational studies had a higher mean age than those in randomized controlled trials; however, the proportion of male patients was slightly lower in observational studies. In randomized controlled trials, the daily doses of NCs ranged from 2 mg to 8 mg; however, daily doses of SCs ranged from 20 mg to 100 mg. Daily doses of NCs in observational studies ranged from 0.5 mg to 9 mg, whereas the dose of SC was 50 mg (SCs doses were converted to the corresponding prednisone dose) (Supplementary File S1).

Studies showed relevant outcomes at different time points. For example, Xiao et al. (Nemagouda, 2014; Xiao et al., 2020) reported statistics on day 5, Gunen et al. (Zhou and Han, 2004; Gunen et al., 2007; Zhang et al., 2011; Gong et al., 2013; Sun et al., 2014; Wang and Chen, 2014; Ding et al., 2016; Zhao et al., 2019; Jiang, 2020; Gu, 2021; Liao et al., 2021) measured outcomes on day 7, Kafee et al. (Maltais et al., 2002; Mirici et al., 2003; Kafee et al., 2016; Zhao, 2016; Shi and Geng, 2018) measured outcomes on day 10, Chen et al. (Song and Hu, 2015; Zheng et al., 2019; Chen et al., 2020) measured outcomes after day 10, and Yilmazel Ucar et al. (Yilmazel Ucar et al., 2014) did not perform measurements until asymptomatic discharge (Supplementary File S1).

### 3.2 Risk of bias

**FIGURE 2:**
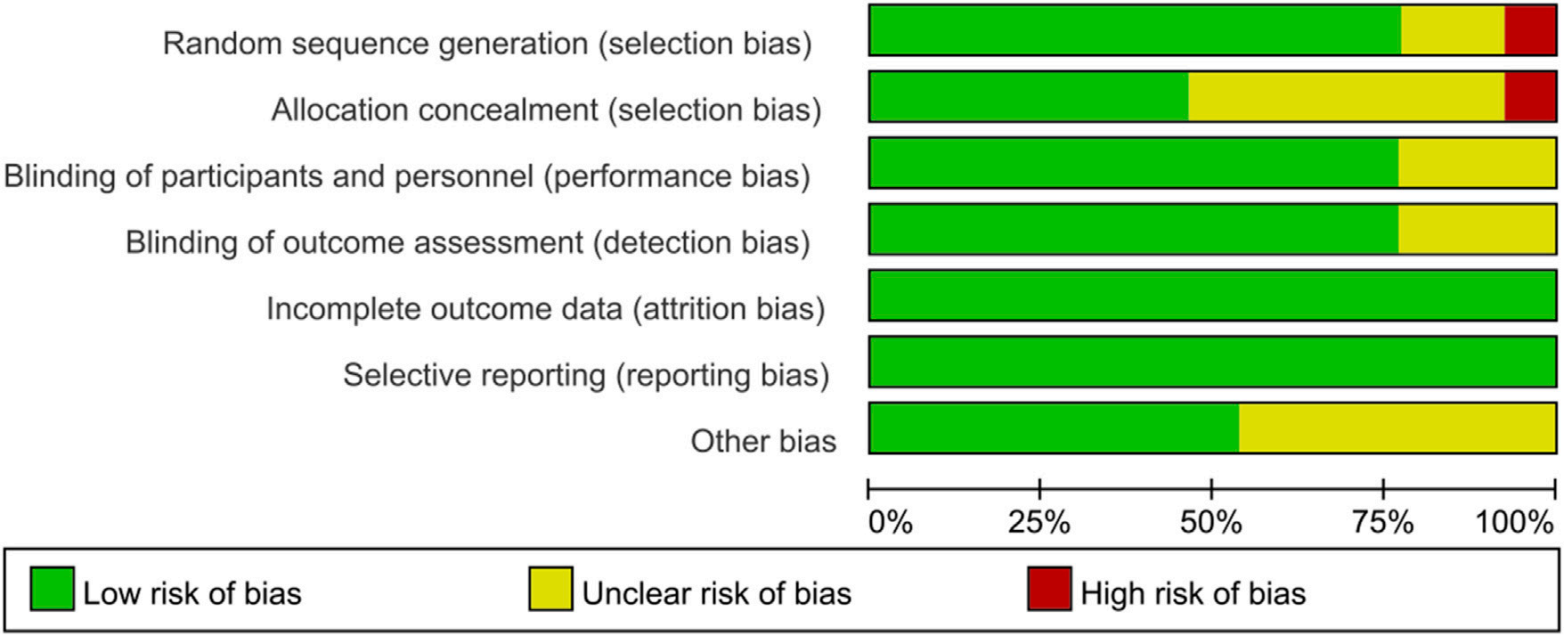
Cochrane Risk of Bias graph.

According to the Cochrane Risk of Bias Tool (Higgins et al., 2011), all 13 included randomized controlled trial studies revealed the patient status before treatment. Six studies clearly used random number tables to generate randomized series. Six studies did not describe allocation concealment. Three studies did not describe whether they blinded to investigators and participants. Ten studies mentioned the blinding of outcome assessment. In seven studies, no other bias was found. No studies showed evidence of reporting bias. All articles reported outcomes in detail and considered missing patients (no patients were missing). The results of the risk of bias evaluation of the included studies are shown in Figure 2. Overall, randomization was present in most studies with an acceptable level of bias.

No high-risk bias items were identified in all included observational studies. The Newcastle-Ottawa Scale is provided in Supplementary File S1 and is available online. Overall, the quality of the included observational studies and randomized controlled trials was moderate to very high. The results suggested that the average study quality was acceptable.

### 3.3 Analysis of outcomes

Data heterogeneity at baseline was low in all groups in both types of studies. The respective analyses in each study showed no statistically significant differences between the two groups at baseline (*p* > 0.05). Therefore, direct post-treatment data were used for the analysis.

### 3.4 Comparison of benefits reported by observational studies and randomized controlled trials

#### 3.4.1 Changes in pulmonary function (FEV_1_%pred and FEV_1_)

**FIGURE 3:**
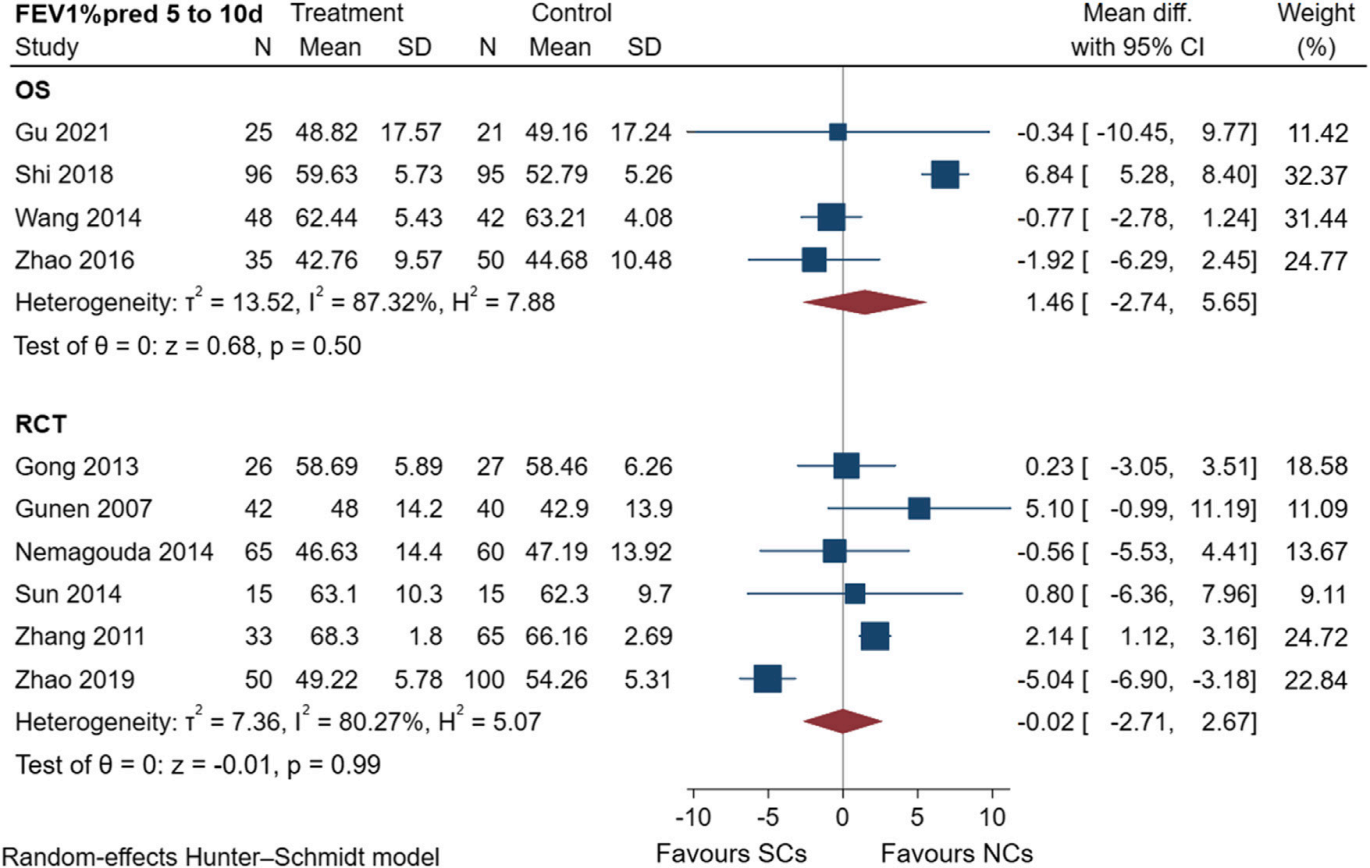
Effectiveness of SCs and NCs on FEV_1_%pred at 5–10 days control in observational studies and randomized controlled trials.

FEV_1_%pred scores of four observational studies (Wang and Chen, 2014; Zhao, 2016; Shi and Geng, 2018; Gu, 2021) and six randomized controlled trials (Gunen et al., 2007; Zhang et al., 2011; Gong et al., 2013; Nemagouda, 2014; Sun et al., 2014; Zhao et al., 2019) are shown in Figure 3, including a total of 950 patients at the study endpoint (days 5–10). The post-treatment FEV_1_%pred levels were heterogeneous among the studies (observational studies: I^2^ = 87.32%, *p* = 0.50; randomized controlled trials: I^2^ = 80.27%, *p* = 0.99). Based on the set subgroups for analysis, heterogeneities in observational studies were related to the number of patients and sex ratio (Supplementary File S1). In a study (Shi and Geng, 2018) with a high effect on heterogeneity, the source of heterogeneity was considered as the differences in the additional treatments, number of patients, and sex ratio. Heterogeneities in randomized controlled trials were related to the dose of NC and the number of patients (Supplementary File S1). The advantage of NCs was evident when NC doses were in the range of 6–8 mg and the number of patients exceeded 100, but the effect of SCs was significant when NC doses were in the range of 4–6 mg and the number of patients was 60–100. The sensitivity analysis suggested stable results (Supplementary File S1). An analysis of FEV_1_%pred data showed that both NCs and SCs had favorable effects on lung function, and the random-effects model showed no significant differences between NCs and SCs in FEV_1_%pred data (*p* > 0.05).

**FIGURE 4:**
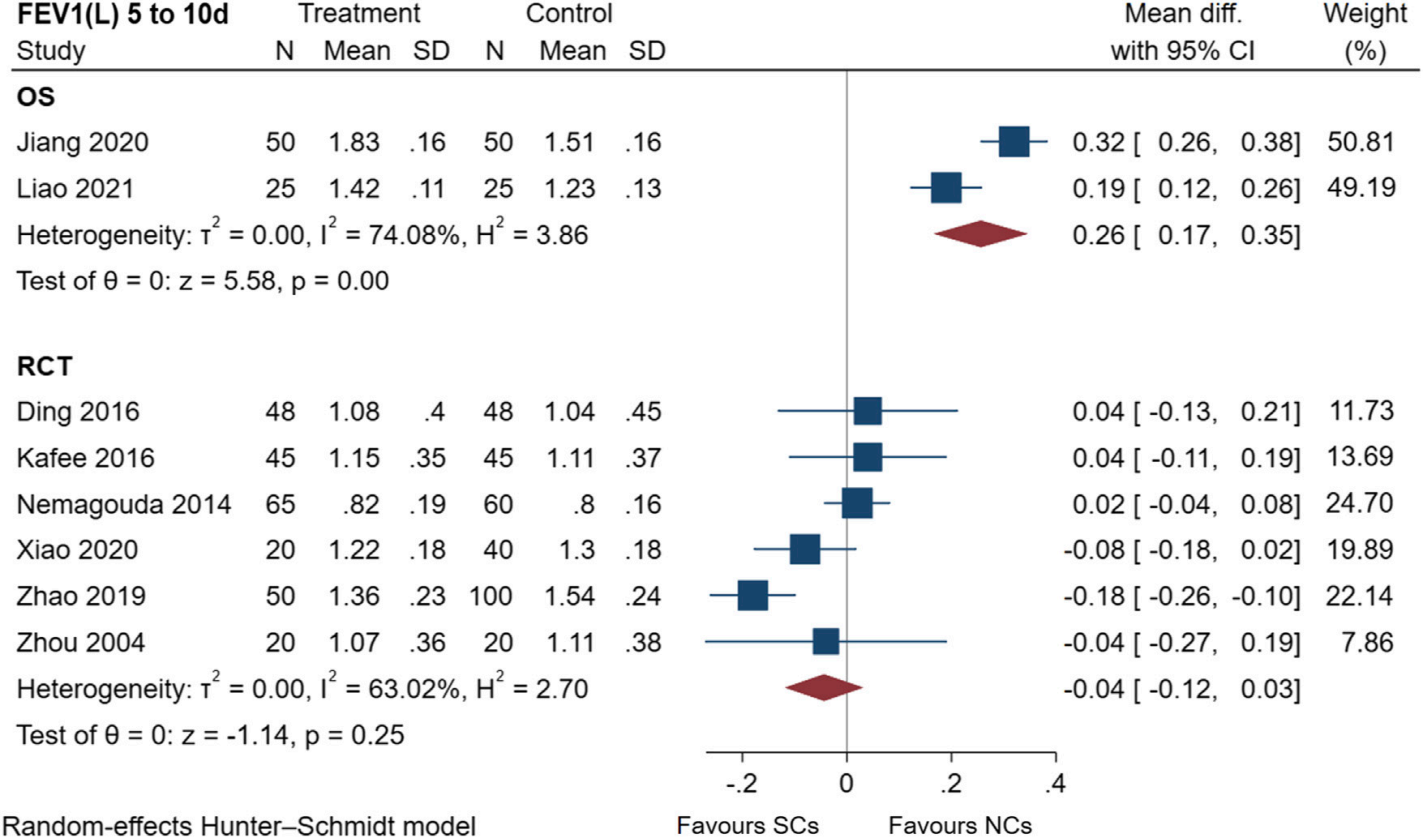
Effectiveness of SCs and NCs on FEV_1_(L) at 5–10 days control in observational studies and randomized controlled trials.

The FEV_1_ results of two observational studies (Jiang, 2020; Liao et al., 2021) and six randomized controlled trials (Zhou and Han, 2004; Nemagouda, 2014; Ding et al., 2016; Kafee et al., 2016; Zhao et al., 2019; Xiao et al., 2020) are presented in Figure 4, including a total of 711 patients at the study endpoint (days 5–10). Post-treatment FEV_1_ levels were heterogeneous between studies (observational studies: I^2^ = 74.08%, *p* < 0.005; randomized controlled trials: I^2^ = 63.02%, *p* = 0.25). Only two observational studies were conducted, hence, no subgroup analysis was performed. According to predetermined subgroups, heterogeneities in randomized controlled trials were related to the number of patients (Supplementary File S1). Furthermore, NCs significantly outperformed SCs when the number of patients was less than 60. The sensitivity analysis suggested stable results (Supplementary File S1). An analysis of FEV_1_ data showed that both NCs and SCs had a favorable effect on lung function, whereas the combined results of the FEV_1_ studies demonstrated differences between the two groups (NCs and SCs). A significant difference (*p* < 0.005) was found in observational studies.

#### 3.4.2 Change in blood gas analysis results (PaO_2_, PaCO_2_, and SaO_2_)

**FIGURE 5:**
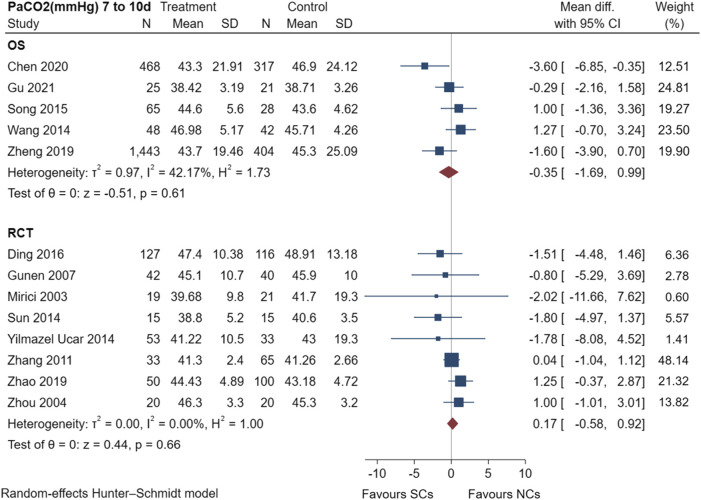
Effectiveness of SCs and NCs on PaCO_2_ (mmHg) at 7–10 days control in observational studies and randomized controlled trials.

Figure 5 shows the results of the examination of PaCO_2_ in five observational studies (Wang and Chen, 2014; Song and Hu, 2015; Zheng et al., 2019; Chen et al., 2020; Gu, 2021) and eight randomized controlled trials (Mirici et al., 2003; Zhou and Han, 2004; Gunen et al., 2007; Zhang et al., 2011; Sun et al., 2014; Yilmazel Ucar et al., 2014; Ding et al., 2016; Zhao et al., 2019), including a total of 3,630 patients at the study endpoint (days 7–10). Post-treatment PaCO_2_ levels had low heterogeneity in observational studies (I^2^ = 42.17%, *p* = 0.61), but no heterogeneity in randomized controlled trials (I^2^ = 0.00%, *p* = 0.66). The sensitivity analysis suggested stable results (Supplementary File S1). The results of the random-effects model demonstrated that both NCs and SCs had favorable effects on PaCO_2_; however, the differences between PaCO_2_ in the two groups were not statistically significant (*p* > 0.05).

**FIGURE 6:**
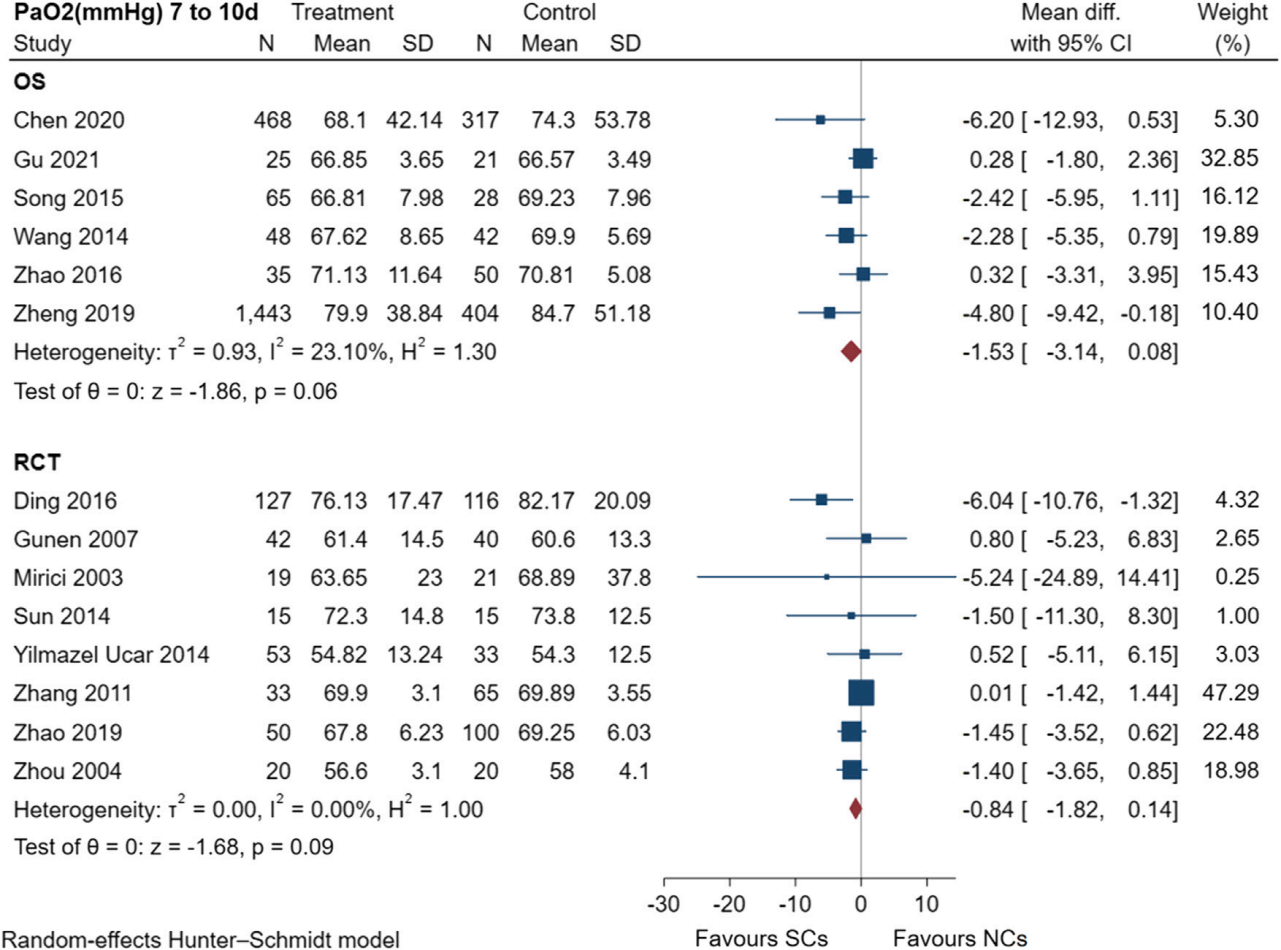
Effectiveness of SCs and NCs on PaO_2_ (mmHg) at 7–10 days control in observational studies and randomized controlled trials.

The PaO_2_ results of six observational studies (Wang and Chen, 2014; Song and Hu, 2015; Zhao, 2016; Zheng et al., 2019; Chen et al., 2020; Gu, 2021) and seven randomized controlled trials (Mirici et al., 2003; Zhou and Han, 2004; Gunen et al., 2007; Zhang et al., 2011; Sun et al., 2014; Ding et al., 2016; Zhao et al., 2019) are shown in Figure 6, including a total of 3,715 patients at the study endpoint (days 7–10). Post-treatment PaO_2_ levels were very low heterogeneous across studies (observational studies: I^2^ = 23.10%, *p* = 0.06; randomized controlled trials: I^2^ = 0.00%, *p* = 0.09). The sensitivity analysis suggested stable results (Supplementary File S1). The results of the random-effects model showed favorable effects of both NCs and SCs on PaO_2_; however, the combined PaO_2_ results of both study types showed no statistically significant difference between groups (*p* > 0.05).

**FIGURE 7:**
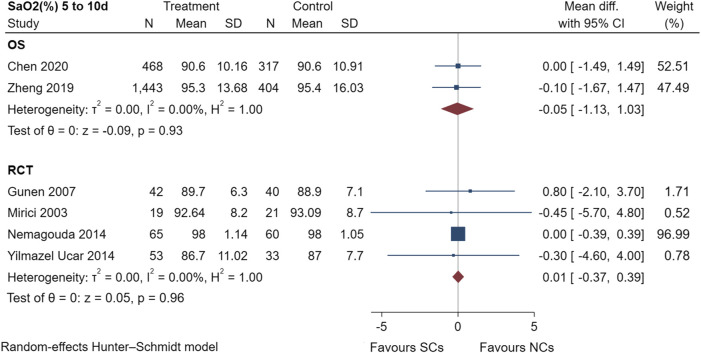
Effectiveness of SCs and NCs on SaO_2_ (%) at 5–10 days control in observational studies and randomized controlled trials.

Figure 7 shows the SaO_2_ results of two observational studies (Zheng et al., 2019; Chen et al., 2020) and four randomized controlled trials (Mirici et al., 2003; Gunen et al., 2007; Nemagouda, 2014; Yilmazel Ucar et al., 2014), including a total of 2,965 patients at the study endpoint (days 5–10). Post-treatment SaO_2_ levels were not heterogeneous across studies (observational studies: I^2^ = 0.00%, *p* = 0.93; randomized controlled trials: I^2^ = 0.00%, *p* = 0.96). The sensitivity analysis suggested stable results (Supplementary File S1). The results of the random-effects model indicated that both NCs and SCs had favorable effects on blood gases; however, the combined SaO_2_ results of both study types showed no statistically significant difference between groups (*p* > 0.05).

**FIGURE 8:**
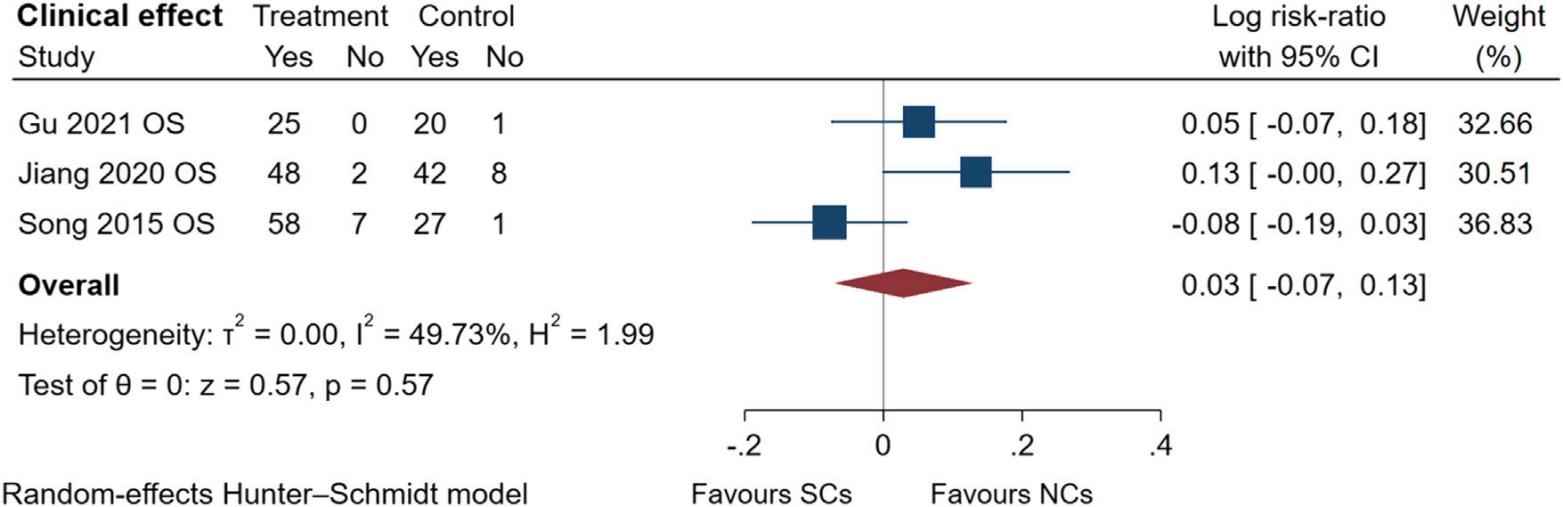
Clinical effect of SCs and NCs in observational studies and randomized controlled trials.

The comparison of clinical effectiveness was derived from three observational studies (Song and Hu, 2015; Jiang, 2020; Gu, 2021) (Figure 8) and low heterogeneity in these studies (I^2^ = 49.73%, *p* = 0.57). NCs and SCs can largely resolve patients’ clinical symptoms, such as cough and chest tightness. The symptoms of AECOPD were partially relieved, and pulmonary ventilation function and blood gas indexes significantly improved compared with those before treatment. Sensitivity analysis confirmed its stability, but data from randomized controlled trials were lacking.

### 3.5 Comparison of adverse events reported by observational studies and randomized controlled trials

**FIGURE 9:**
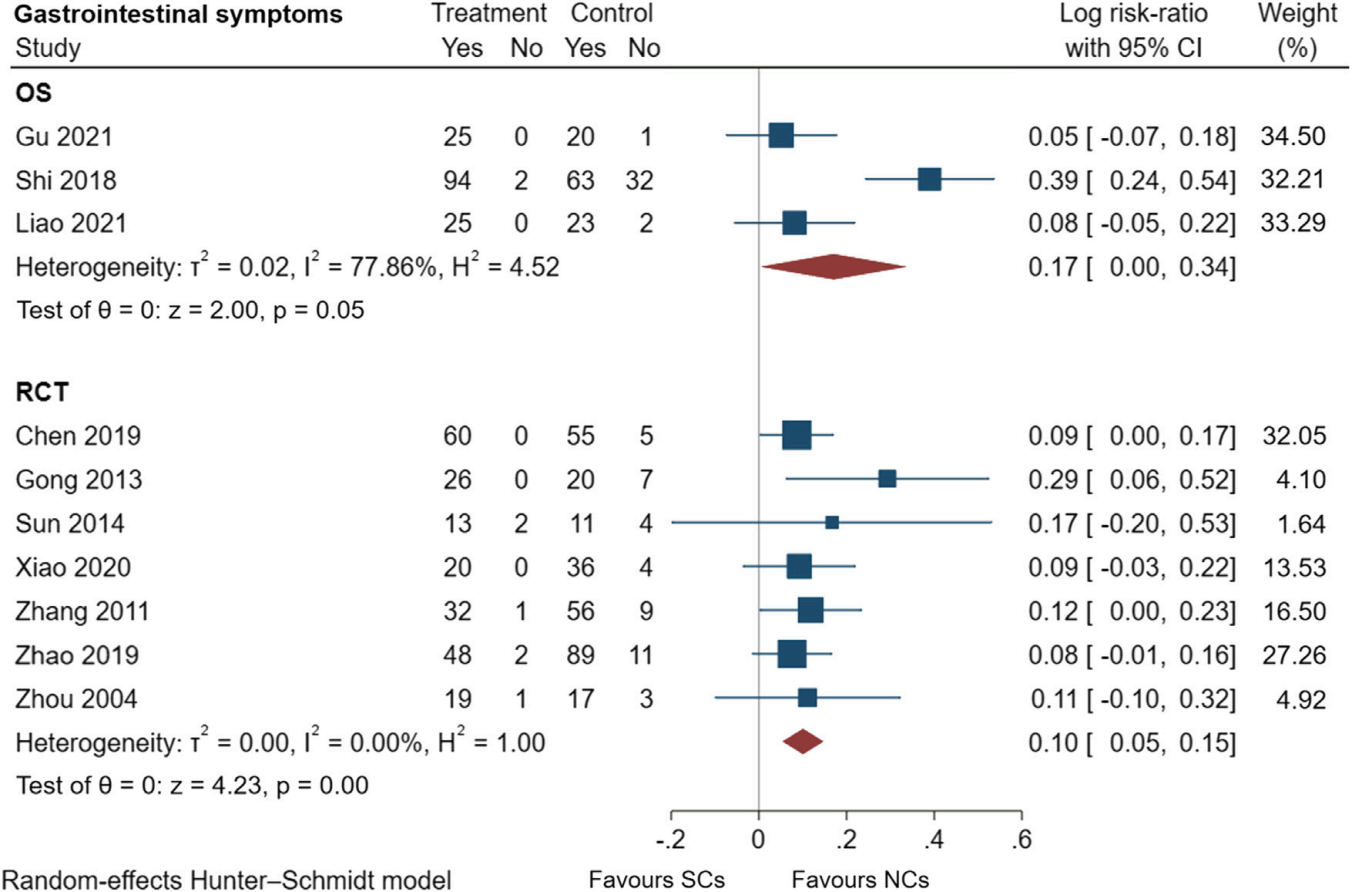
Gastrointestinal symptoms of SCs and NCs in observational studies and randomized controlled trials.

**FIGURE 10:**
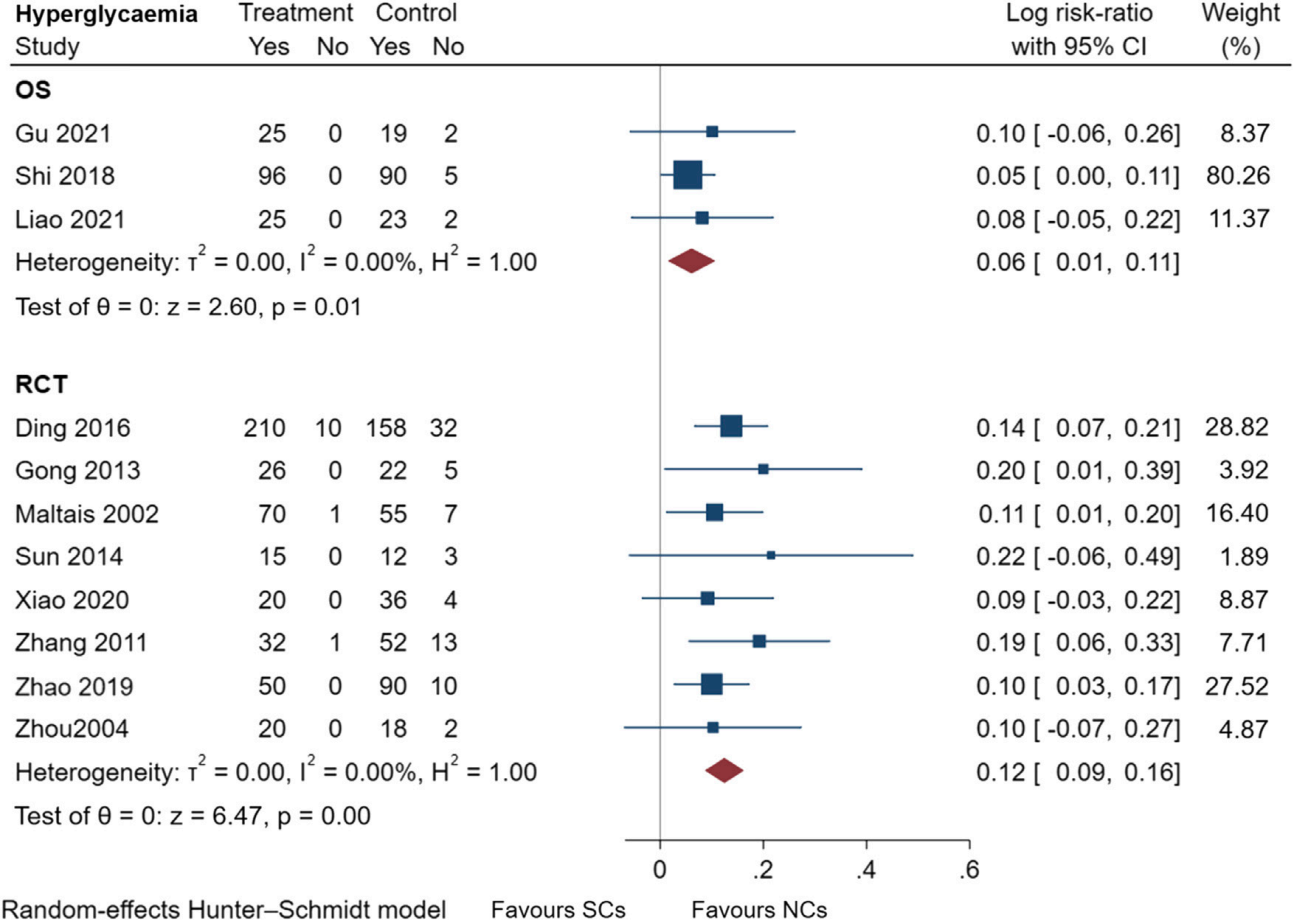
Hyperglycemia events of SCs and NCs in observational studies and randomized controlled trials.

**FIGURE 11:**
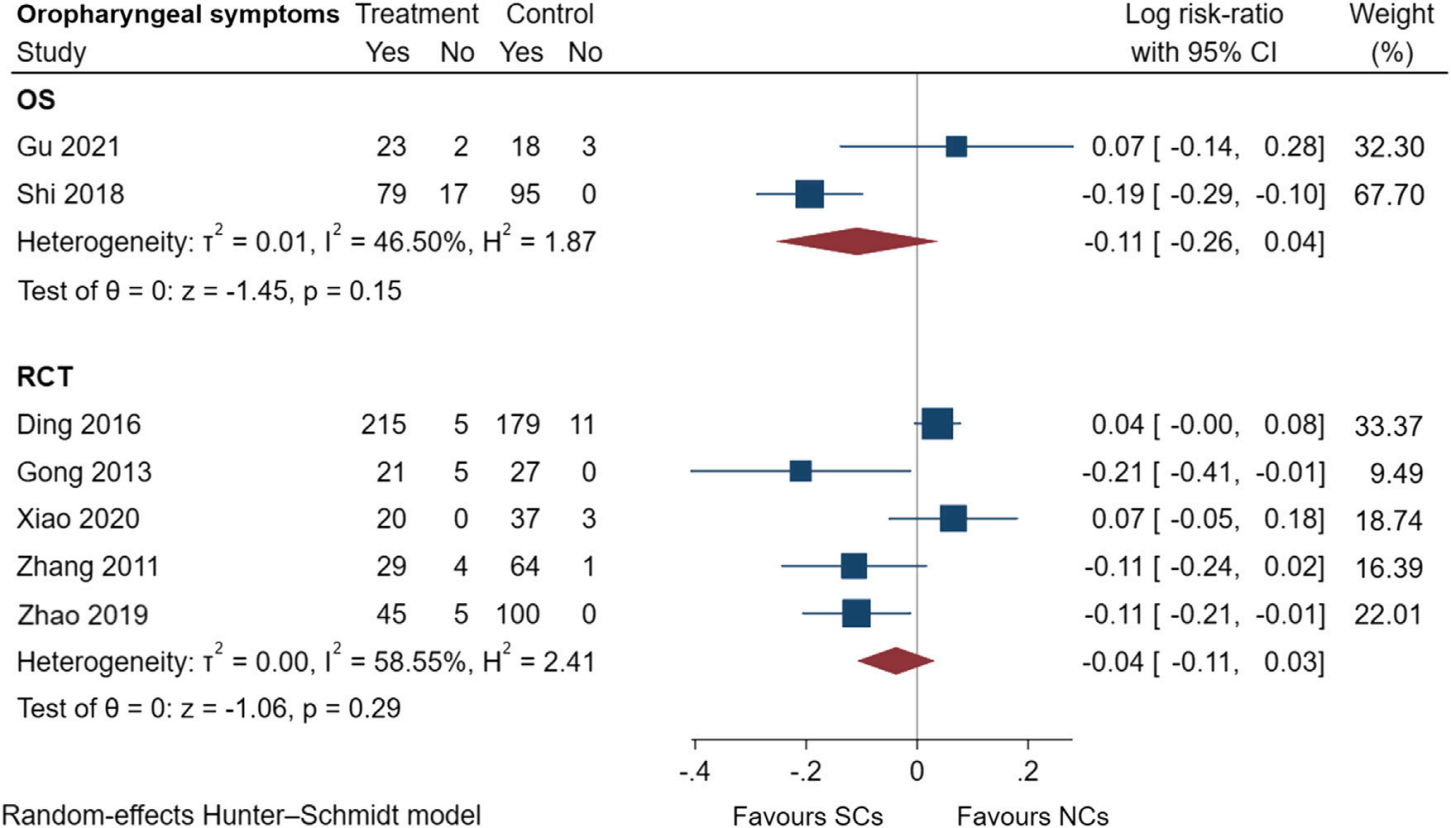
Oropharyngeal symptoms of SCs and NCs in observational studies and randomized controlled trials.

**FIGURE 12:**
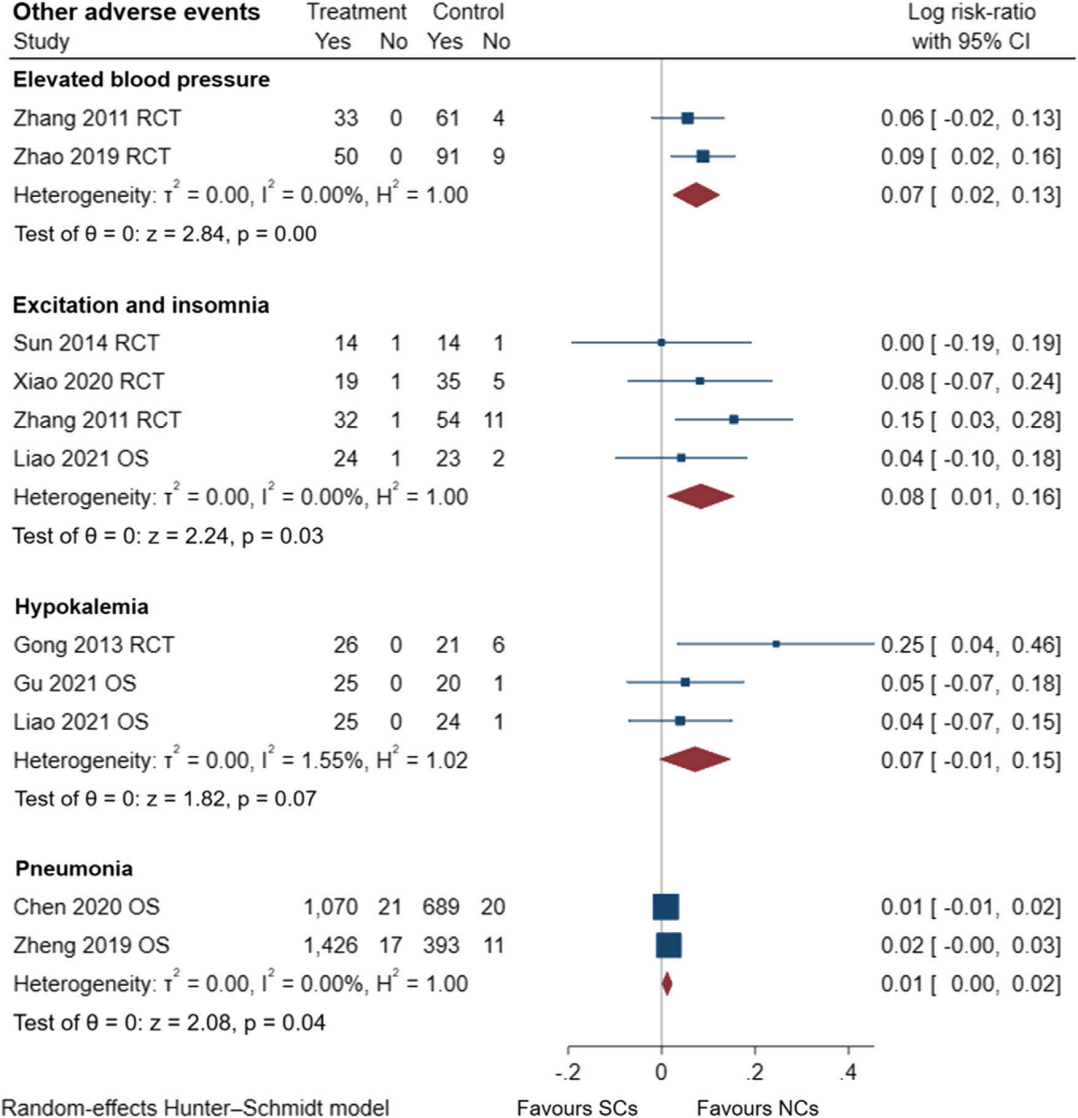
Other adverse events of SCs and NCs in observational studies and randomized controlled trials.

**FIGURE 13:**
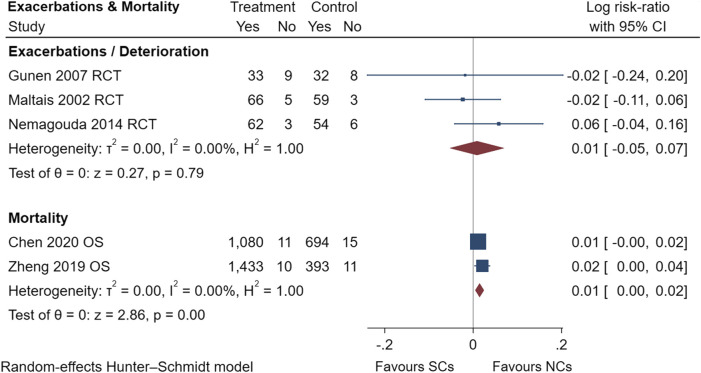
Exacerbations and mortality of SCs and NCs in observational studies and randomized controlled trials.

Figures 9–13 comprehensively analyze the adverse events that occurred with NCs in COPD patients. Adverse reaction types were reported in two or more papers before they were entered into the analysis to prevent the occurrence of incidental events. All events were derived from five observational studies (Shi and Geng, 2018; Zheng et al., 2019; Chen et al., 2020; Gu, 2021; Liao et al., 2021) and ten randomized controlled studies (Maltais et al., 2002; Zhou and Han, 2004; Gunen et al., 2007; Zhang et al., 2011; Gong et al., 2013; Nemagouda, 2014; Sun et al., 2014; Ding et al., 2016; Zhao et al., 2019; Xiao et al., 2020), including a total of 4,990 patients. The adverse reactions, including worsening with treatment, were counted and compared. There was no heterogeneity for most adverse reactions (I^2^ < 50%) and the sensitivity analysis suggested stable results (Supplementary File S1).

Gastrointestinal symptoms included nausea and vomiting, gastric/duodenal ulcer, gastric bleeding, *etc.* Oropharyngeal symptoms included dry mouth, throat discomfort, dental ulcer, oral fungal infection, *etc.* Compared to treatment with NCs, that with SCs significantly increased the risks of hyperglycemia (LogRR, 0.06; 95% CI, 0.01–0.11; I^2^ = 0.00%, *p* = 0.01), pneumonia (LogRR, 0.01; 95% CI, 0.00–0.02; I^2^ = 0.00%, *p* = 0.04), and mortality (LogRR, 0.01; 95% CI, 0.00–0.02; I^2^ = 0.00%, *p* < 0.005) in observational studies. However, there were no significant differences in adverse effects such as gastrointestinal symptoms, oropharyngeal symptoms, excitation and insomnia, and hypokalemia. In randomized controlled trials, treatment with SCs was associated with significantly more gastrointestinal symptoms (LogRR, 0.10; 95% CI, 0.05–0.15; I^2^ = 0.00%, *p* < 0.005), hyperglycemia (LogRR, 0.12; 95% CI, 0.09–0.16, I^2^ = 0.00%, *p* < 0.005), elevated blood pressure (LogRR, 0.07; 95% CI, 0.02–0.13; I^2^ = 0.00%, *p* < 0.005), and hypokalemia (LogRR, 0.25; 95% CI, 0.04–0.46). However, treatment with NCs or SCs showed no significant differences in oropharyngeal symptoms, excitation and insomnia, and exacerbations.

### 3.6 Subgroup analysis based on age, dosage, follow-up time, number of patients, and sex ratio

When the results of the different subgroup analyses were considered together, heterogeneity may depended mainly on Dose of NC, follow-up time, and the number of patients in individual trials. Studies with many patients included simultaneous multicenter data; however, the baseline levels might have differed at each center, thus causing large heterogeneity. The indicators measured at different follow-up times might be influenced by the cumulative effect of the drug or the speed of onset of action, resulting in heterogeneity.

When subgroup analyses were performed by age, SC types, or sex ratio, we observe little heterogeneity in clinical effectiveness and adverse events, except in FEV_1_% pred and gastrointestinal symptoms. Changes in the dose of NC or SC did not result in statistically significant changes in blood gas analysis when analyzed separately for observational studies or randomized controlled trials or uniformly, regardless of study type. The same was true regarding follow-up time, with NCs or SCs having comparable treatment advantages at 7–10 days of detection time in overall patients. Interestingly, the FEV_1_% pred results showed that with the daily dosage of NC increased (4–6 mg to 6–8 mg), the therapeutic advantage shifted from NCs to SCs regardless of study type (Supplementary File S1); however, the effect was as good in the high-dose NC group (>8 mg) as it was in the SC group. Whether this suggests that FEV_1_% pred is a more sensitive indicator for treatment is debatable and deserves further discussion.

### 3.7 Sensitivity analysis and publication bias

We excluded each of the included studies for sensitivity analysis and observed significant changes in outcomes, confirming the primary outcome’s robustness (Supplementary File S1). For publication bias, no potential bias was observed by performing Begg’s test, but Egger’s test identified a publication bias associated with Gastrointestinal symptoms in randomized controlled trials (Supplementary File S1). However, validation by the trim and fill method showed that no additional studies were needed, which indicated the stability of the results. Meanwhile, no funnel plot test was performed, as no particular study outcome included more than 10 studies.

## 4 Discussion

Corticosteroids account for a large proportion of AECOPD treatments. In a study from China, in a medication analysis of 432 eligible AECOPD patients, the proportion of glucocorticoids applied increased with hospital grade (18.6% vs. 45.6% vs. 69.2%; *p* < 0.001) (Liu et al., 2022). A cross-sectional study by the Veterans Health Administration in 2020 found that 23.9% of 26,536 patients with COPD without a history of severe or frequent exacerbations and without airflow disorders were treated with inhaled corticosteroids (Griffith et al., 2020). The use of inhalation agents are increasing in proportion, but nebulization can be more uniform and less irritating than inhalation administration, with higher local activity and rapid hepatic metabolism (Szefler, 2001). In the included studies, budesonide was selected as the drug in the experimental group. Budesonide’s non-classical pathway and its strong hydrophilicity (higher than beclomethasone dipropionate and fluticasone propionate) allow it to penetrate the airway mucus layer more quickly and exert its efficacy.

This work showed that NCs were comparable to SCs in improving lung function and blood gas parameters. Comparing their treatment effects did not reveal significant differences between randomized controlled trials and observational studies. Furthermore, NCs were associated with a lower incidence of hyperglycemia, gastrointestinal symptoms, excitation and insomnia, and elevated blood pressure than SCs. The results of our meta-analysis support the conclusion that NCs can be a suitable alternative to SCs for treating AECOPD.

The pooled effect of three previous meta-analyses (Zhai et al., 2017; Pleasants et al., 2018; Gu et al., 2020) indicated no significant differences in pulmonary function and blood gas changes in patients with AECOPD when using different treatment regimens (inhaled corticosteroids *versus* SCs). Although our analysis shared similarities with previous analyses, we further refined it to complement the new randomized controlled trial and observational studies while excluding poor quality literature. First, different from the previous meta-analysis by Zhai et al. (Zhai et al., 2017), our study added some newly reported results, including FEV_1_, all adverse events, exacerbations, and mortality; therefore, our results were more reliable. Additionally, unlike the previous meta-analysis by Pleasants et al. (Pleasants et al., 2018) and Gu et al. (Gu et al., 2020), our analysis included a total of 13 randomized controlled trial studies and a larger real-world sample, which advantageously extrapolated the conclusions in real life. Our analysis also excluded three trials (Han and Zhou, 2004; Chen et al., 2005; Ställberg et al., 2009) because their experimental design was flawed, with insufficient randomization and lack of complete data.

Additionally, our analysis assessed more metrics than the previous three meta-analyses and performed subgroup analyses according to sex, age, population, and dose when necessary (I^2^ > 50%). Therefore, our results can be considered more comprehensive and reliable. Overall, based on the available evidence, our meta-analysis noted that NCs improved FEV_1_%pred and FEV_1_ is similar to SCs in the randomized controlled trials. However, observational studies noted that NCs improved FEV_1_ significantly better than SCs at 5–10 days after treatment. Regarding arterial blood gas results, there were no differences between NCs and SCs in terms of PaO_2_, PaCO_2_, and SaO_2_ values at 7–10 days after treatment. In terms of adverse events, both the randomized controlled trials and observational studies noted a significant difference in the incidence of hyperglycemia between treatments with NCs and SCs, with NCs being less likely to cause elevated blood glucose levels. The same was true for gastrointestinal symptoms, which were less frequent with NCs than with SCs. Additionally, treatment with SCs caused elevated blood pressure, excitation and insomnia; their incidence was statistically significantly different from that of NCs.

Using a subgroup analysis, we found that NC dose of 4–6 mg was advantageous because it improved the FEV_1_% pred results regardless of study type, which could be reliably demonstrated. In contrast, the dominance of SC predominated when the dose of NC was 6–8 mg. Furthermore, the positive effect of the dose of NCs or SCs on blood gas analysis results remained consistent. A meta-analysis by Gu et al. (Gu et al., 2020) found that nebulized inhalation of budesonide had a greater benefit than SCs for SaO_2_ at 48–72 h after treatment; however, this benefit disappeared at 5–7 days after treatment. Since our follow-up time for SaO_2_ was 5–10 days after treatment, no advantage of NCs was found.

There are limitations to our meta-analysis. First, according to our inclusion criteria, we included a total of 22 trials. Although 5,764 participants were included, the real-world data for many patients has heterogeneous sources and the full accuracy of the results cannot be guaranteed. Second, none of the cohort studies of observational studies reported long-term follow-up data, which may be related to the rapid onset and short duration of AECOPD; therefore, the long-term efficacy and safety of NCs after treatment compared with SCs are unclear. Finally, the studies included in this meta-analysis varied considerably of doses of NCs, making it impossible to give an optimal dose group. The advantages of NCs would be more obvious if future studies provide insight regarding whether sequential treatment or different dosages would be more beneficial.

In conclusion, compared with treatment with NCs, SCs elevated the risks of gastrointestinal symptoms by 11% and a 7% increased risk of elevated blood pressure in randomized controlled trials. SCs increased the risks of hyperglycemia by 6% and 13% in observational studies and randomized controlled trials, respectively. SCs also elevated the risks of excitation and insomnia by 8% in patients with AECOPD, regardless of study type. Furthermore, the patient’s pulmonary function and blood gas analysis results improved rapidly and to a comparable extent. NCs are worthy of clinical promotion and have comparable efficacy to SCs. The consistency of observational studies and randomized controlled trials was also validated in this study, which facilitates the use and emphasis on real-world evidence. Due to limitations of our analysis, further studies are necessary to validate these results.

## Data Availability

The original data supporting the conclusions of this article will be provided by the authors without reservation.
