# Supplementary material for: Nebulized corticosteroids versus systemic corticosteroids for patients with acute exacerbation of chronic obstructive pulmonary disease: A systematic review and meta-analysis comparing the benefits and harms reported by observational studies and randomized controlled trials
Source: Front Pharmacol. 2022 Oct 5;13:966637. doi: 10.3389/fphar.2022.966637 (PMC9581123; doi:10.3389/fphar.2022.966637)
Supplement: Supplementary file 1 [file DataSheet1.pdf]

SUPPLEMENTAL FILE 1

Title: Nebulized corticosteroids versus systemic corticosteroids for patients with acute exacerbation of chronic obstructive pulmonary disease: A systematic review and meta-analysis comparing the benefits and harms reported by observational studies and randomized controlled trials

Contents

Tables

Supplementary Table 1. Search strategy used in April 1, 2022....04

Supplementary Table 2. Characteristics of the included RCTs....07

Supplementary Table 3. Characteristics of the included OSs....09

Supplementary Table 4. Subgroup characteristics of the included Studies....11

Supplementary Table 5. Newcastle-Ottawa Quality Assessment Scale for Cohort Studies....12

Supplementary Table 6. Publication Bias in outcome of interest....13

Figures

Supplementary Figure 1. Sensitivity analysis of FEV_1% pred at 5 to 10d control in OSs and RCTs....14

Supplementary Figure 2. Sensitivity analysis of FEV_1 (L) at 5 to 10d control in OSs and RCTs....14

Supplementary Figure 3. Sensitivity analysis of $PaCO_2$ (mmHg) at 7 to 10d control in OSs and RCTs....15

Supplementary Figure 4. Sensitivity analysis of PaO_2 (mmHg) at 7 to 10d control in OSs and RCTs..........15
Supplementary Figure 5. Sensitivity analysis of SaO_2 (%) at 5 to 10d control in OSs and RCTs..........16
Supplementary Figure 6. Sensitivity analysis of clinical effect in OSs..........16
Supplementary Figure 7. Sensitivity analysis of Gastrointestinal symptoms in OSs and RCTs..........17
Supplementary Figure 8. Sensitivity analysis of Hyperglycemia in OSs and RCTs..........17
Supplementary Figure 9. Sensitivity analysis of Oropharyngeal symptoms in OSs and RCTs..........18
Supplementary Figure 10. Sensitivity analysis of a) Hypokalemia, b) Pneumonia in OSs..........18
Supplementary Figure 11. Sensitivity analysis of a) Elevated blood pressure, b) Excitation and insomnia in RCTs..........19
Supplementary Figure 12. Sensitivity analysis of a) Mortality, b) Exacerbations / deterioration in OSs and RCTs..........19
Supplementary Figure 13. Subgroup (Number of patients, Sex ratio) analysis of FEV1% pred at 5 to 10d control in OSs..........20
Supplementary Figure 14. Subgroup (Dose of NC, Number of patients) analysis of FEV1% pred at 5 to 10d control in RCTs..........21
Supplementary Figure 15. Subgroup (Dose of NC) analysis of FEV_1 % pred at 5 to 10d control in OSs and RCTs..........22
Supplementary Figure 16. Subgroup (Number of patients) analysis of FEV_1(L) at 5 to 10d control in RCTs..........23
Supplementary Figure 17. Subgroup (Dose of NC, Follow-up time, Number of patients) analysis of Gastrointestinal symptoms in OSs..........24
Supplementary Figure 18. Subgroup (Age, Follow-up time) analysis of Oropharyngeal symptoms in RCTs..........25
Supplementary Figure 19. Trim and fill method of Gastrointestinal symptoms in RCTs..........26

Abbreviations in Supplementary File

CAT: Chronic obstructive pulmonary disease assessment test

FEV_1: Forced expiratory volume in the first second

FVC: Forced vital capacity

NCs: Nebulized corticosteroids

NR: Not reported

OSs: Observational studies

$PaCO_2$: Partial pressure of carbon dioxide in artery

PaO_2: Partial pressure of oxygen in artery

RCT: Randomized controlled trial

SCs: Systemic corticosteroids

SaO_2: Oxygen saturation

Supplementary Table 1. Search strategy used in April 1，2022

EMBASE Search strategy

**Search steps**	**Search strategy**
#1	'chronic obstructive lung disease'/exp
#2	('chronic obstructive lung disease' OR 'pulmonary disease, chronic obstructive' OR 'chronic obstructive pulmonary disease*' OR 'coad' OR 'copd' OR 'chronic obstructive airway disease' OR 'airflow obstruction, chronic' OR 'chronic airflow obstruction'):ti,ab,kw
#3	'acute exacerbation of chronic obstructive pulmonary disease'/exp
#4	('acute exacerbation of chronic obstructive pulmonary disease' OR 'acute exacerbation of copd' OR 'acute copd exacerbation' OR 'aecopd' OR 'ae copd'):ti,ab,kw
#5	#1 OR #2 OR #3 OR #4
#6	('beclometasone' OR 'fluticasone' OR 'budesonide')/exp
#7	('beclometasone' OR 'beclomethasone' OR 'fluticasone' OR 'budesonide' OR 'nebulized corticosteroid' OR 'inhaled corticosteroids'):ti,ab,kw
#8	#6 OR #7
#9	('betamethasone' OR 'methylprednisolone' OR 'prednisolone' OR 'hydrocortisone' OR 'prednisone' OR 'dexamethasone')/exp
#10	('betamethasone' OR 'methylprednisolone' OR 'prednisolone' OR 'hydrocortisone' OR 'prednisone' OR 'dexamethasone' OR 'systemic corticosteroid' OR 'oral corticosteroid' OR 'intravenous corticosteroid'):ti,ab,kw
#11	#9 OR #10
#12	#5 AND #8 AND #11
Items found	865

MEDLINE Search strategy

Search steps	Search strategy
1	TS=(Pulmonary Disease, Chronic Obstructive OR Chronic Obstructive Lung Disease OR Chronic Obstructive Pulmonary Diseases OR COAD OR COPD OR Chronic Obstructive Airway Disease OR Chronic Obstructive Pulmonary Disease OR Airflow Obstruction, Chronic OR Airflow Obstructions, Chronic OR Chronic Airflow Obstructions OR Chronic Airflow Obstruction OR Acute Exacerbation of Chronic Obstructive Pulmonary Disease OR Acute Exacerbation of COPD OR Acute COPD Exacerbation OR AECOPD OR ae copd)
2	TS=(Beclomethasone OR Fluticasone OR Budesonide OR Nebulized Corticosteroid* OR Inhaled Corticosteroid*)
3	TS=(Betamethasone OR Methylprednisolone OR Prednisolone OR Hydrocortisone OR Prednisone OR Dexamethasone OR Systemic Corticosteroid* OR Oral Corticosteroid* OR Intravenous Corticosteroid*)
4	#1 AND #2 AND #3
Items found	1219

The Cochrane Library Search strategy

Search steps	Search strategy
#1	MeSH descriptor: [Pulmonary Disease, Chronic Obstructive] explode all trees
#2	(Pulmonary Disease, Chronic Obstructive):ti,ab,kw OR (Chronic Obstructive Lung Disease):ti,ab,kw OR (Chronic Obstructive Pulmonary Diseases):ti,ab,kw OR (COAD):ti,ab,kw OR (COPD):ti,ab,kw (Word variations have been searched)
#3	(Chronic Obstructive Airway Disease):ti,ab,kw OR (Chronic Obstructive Pulmonary Disease):ti,ab,kw OR (Airflow Obstruction, Chronic):ti,ab,kw OR (Airflow Obstructions, Chronic):ti,ab,kw OR (Chronic Airflow Obstruction):ti,ab,kw (Word variations have been searched)
#4	(Acute Exacerbation of Chronic Obstructive Pulmonary Disease):ti,ab,kw OR (Acute Exacerbation of COPD):ti,ab,kw OR (Acute COPD Exacerbation):ti,ab,kw OR (AECOPD):ti,ab,kw OR (ae copd):ti,ab,kw (Word variations have been searched)
#5	#1 OR #2 OR #3 OR #4
#6	MeSH descriptor: [Beclomethasone] explode all trees
#7	MeSH descriptor: [Fluticasone] explode all trees
#8	MeSH descriptor: [Budesonide] explode all trees
#9	(Beclomethasone):ti,ab,kw OR (Fluticasone):ti,ab,kw OR (Budesonide):ti,ab,kw OR (Nebulized Corticosteroid):ti,ab,kw OR (Inhaled Corticosteroid):ti,ab,kw (Word variations have been searched)
#10	#6 #7 OR #8 OR #9
#11	MeSH descriptor: [Betamethasone] explode all trees
#12	MeSH descriptor: [Methylprednisolone] explode all trees
#13	MeSH descriptor: [Prednisolone] explode all trees
#14	MeSH descriptor: [Hydrocortisone] explode all trees
#15	MeSH descriptor: [Prednisone] explode all trees
#16	MeSH descriptor: [Dexamethasone] explode all trees
#17	(Betamethasone):ti,ab,kw OR (Methylprednisolone):ti,ab,kw OR (Prednisolone):ti,ab,kw OR (Hydrocortisone):ti,ab,kw OR (Prednisone):ti,ab,kw (Word variations have been searched)
#18	(Dexamethasone):ti,ab,kw OR (Systemic Corticosteroid):ti,ab,kw OR (Oral Corticosteroid):ti,ab,kw OR (Intravenous Corticosteroid):ti,ab,kw (Word variations have been searched)
#19	#11 OR #12 OR #13 OR #14 OR #15 OR #16 OR #17 OR #18
#20	#5 AND #10 AND #19
Items found	625

Supplementary Table 2. Characteristics of the included Randomized Controlled Trials studies

Study (year)	Study design	Duration of treatment	Composition of patients	No. of patients (NCs/SCs)	Sex (M:F)		Age (year)		Treatment comparisons		Additional treatments	Outcomes of interest
					NCs	SCs	NCs	SCs	NCs	SCs		
Ding et al (2016)	RCT	Until discharge	Inpatient	471(233/238)	178:42	154:36	73.49±8.61	73.18±8.50	Inhalation of budeso-nide 2 mg, 3 times/ day	Injection of methylprednisolone 40 mg/day	Application of bronchodilators, oral or intravenous injection of antibiotics, apophlegmatisants, and controlled oxygen therapy	Change in FEV_1, FEV_1/FVC (%), biochemical parameters at 7 d control; Change in PaO_2, and $PaCO_2$ at 7-10 d Control; CAT score at 24h, 3-4d, 7-10d
Gong et al (2013)	RCT	7 d	Inpatient	53(26/27)	22:4	22:5	62.15±5.45	61.59±5.10	Nebulized budesonide 1 mg 3 times/ Day for 7 d	Injection of methylprednisolone 40 mg/day	Routine treatment of anti-infection, oxygen therapy, bronchodilator (aminophylline, salbutamol), antitussive and expectorant (ambroxol)	Change in FEV_1%, BODE score, Blood-gas analysis at 3 d, 7 d control and adverse events
Gunen et al (2007)	RCT	10-15 d	Inpatients	82(42/40)	35:7	33:7	63.9±9.7	64.9±7.1	Nebulized budesonide 1500 µg four times daily	Prednisolone 40 mg intravenous once daily	Utilise nebulised salbutamol as rescue medication, supplemental oxygen, systemic methylxanthines, and antibiotics were used where signs of bacterial infection existed	Complete blood counts, detailed biochemical analysis, spirometric measurements and arterial blood gas analysis were carried out at admission, 24 h, 72 h, 7 days and 10 days
Kafee et al (2016)	RCT	10 d	Inpatient	90(45/45)	39:6	39:6	60.38±10.34	62.49±10.61	Nebulised budesonide 1mg/dose 12 hourly	Oral prednisolone 40mg/dose at morning	Nebulized β_2 agonists, ipratropium bromide, oral antibiotics, and supplemental oxygen; Theophylline	FEV_1, FVC, FEV_1/FVC, Modified Borg Dyspnoea Score at 3d and 10d
Maltais et al (2002)	RCT	10 d	Inpatients	133(71/62)	57:14	52:10	69.1±8.7	70.4±7.7	Nebulized budesonide 2 mg Q6h for 72 h; Inhaled BUD 2,000 µg/d for 7 d	Oral prednisolone 30 mg Q12h for 72h; 40 mg/d oral prednisolone for 7 d	Terbutaline or salbutamol; Ipratropium bromide; Methylxanthines; Oral antibiotic therapy	Change in post-BD FEV_1, pre-BD FEV_1, dyspnea score, and arterial blood gases from H_0 to H_{72}, duration of hospitalization, and adverse events
Mirici et al (2003)	RCT	10 d	Inpatients	40(19/21)	13:6	16:5	63.06	64.8	Nebulized budesonide 4 mg twice daily	Prednisolone 40 mg intravenous once daily	β-agonist and anticholinergic; aminophylline; antibacterials; oxygen therapy	Change in PaO_2, SaO_2, and $PaCO_2$ at 24 h, 48 h, and 10 d control
Nemagouda et al (2014)	RCT	10 d	Inpatients	130(65/65)	NR	NR	64.2±9.11	62.4±11.31	Budesonide nebulization (2 mg diluted in 4 ml every sixth hourly)	Parenteral / Oral steroids (IV hydrocortisone 200 mg tds / 40 mg of oral prednisolone) once daily	Salbutamol and ipratropium; supplemental oxygen inhalation, deriphyllin; antibiotics	Change in FEV_1, FEV_1%pred, FEV_1/ FVC (%), SaO_2 at 24 h, 72 h, and 5 d control

Sun et al (2014)	RCT	NR	NR	30(15/15)	9:6	8:7	62.8±7.3	62.0±8.1	Inhaled budesonide 3 mg twice daily	Methylprednisolone acetate injectable suspension 40 mg Qd for 3 days then methylprednisolone tablets 8 mg twice daily	LAMA;<br>LABA;<br>antibacterials	Change in PaO_2, $PaCO_2$ at 4 h, 4 d, and 7 d control; Change in FEV_1%pred at 7 d control
Xiao et al (2020)	RCT	5 d	Inpatient	60(20/40)	10:10	(1) 12:8<br>(2) 11:9	58.93±5.86	(1)58.96±4.52<br>(2)58.99±5.77	Nebulized budesonide 1 mg 3 times/ Day for 5 d	(1) Oral prednisone tablets 0.5mg-1.0mg / kg once a day.<br>(2) Prednisolone 40 mg intravenous once daily	Routine treatments such as relieving asthma, resolving phlegm and relieving cough, inhaling oxygen, nutritional support and correcting electrolyte balance	Change in FEV_1 (L), FVC (L), FEV_1/FVC (%), TNF-α, CRP at 5days control and adverse events
Yilmazel Ucar et al (2014)	RCT	Until discharge	Inpatients	86 (53/33)	(1) 25:2<br>(2) 28:4	24:9	(1) 66.7±9.7<br>(2) 69.6±8.5	66.6±9.6	Budesonide<br>(1) 2 mg twice daily<br>(2) 4 mg twice daily	Methylprednisolone 40 mg intravenous once daily	β-agonist and anticholinergic; aminophylline; supplemental oxygen therapy	Change in PaO_2, $PaCO_2$, SaO_2 at 24 h, 48h, and discharge day control
Zhang et al (2011)	RCT	7 d	Inpatients	98(69/29)	23:10	(1)24:10<br>(2) 22:9	66.2	(1) 64.6<br>(2) 65.2	Nebulized budesonide 2 mg 3 times/ Day for 7 d	(1)Oral prednisone tablets 30 mg once a day for 7 days<br>(2)Intravenous methylprednisol-one 80mg once a day for 7 days	Bronchodilators, antibiotics, expectorants, oxygen therapy and correction of acid-base imbalance	Change in pulmonary function test, arterial blood gas analysis, 6-min walking test and quality of life score before treatment at 72h and 7 d control and adverse events
Zhao et al (2019)	RCT	7 d	Inpatient	150(50/100)	29:21	(1)28:22<br>(2)31:19	62.73±5. 91	(1) 63.15±6.20<br>(2) 62.90±5.58	Nebulized budesonide 4 mg twice daily for 7 d	(1) Oral prednisone tablets 40 mg once a day for 7 days<br>(2)Methylprednisolone sodium succinate (specification: 40 mg) 1 mg / (kg d) for injection was given intravenously for 3 days, and the dose was changed to 0.5 mg / (kg d), continuous treatment for 4 days	Routine symptomatic support therapy such as anti-infection, oxygen inhalation, expectorant, relieving asthma and correcting water, electrolyte and acid-base balance	Change in FEV_1, FEV_1 (L), FEV_1% pred, FEV_1/FVC (%), PaO_2, $PaCO_2$, TNF-α, CRP, IL-6 at 7days control and adverse events
Zhou et al (2004)	RCT	7 d	NR	40(20/20)	16:4	17:3	62.9±6.4	67.2±6.2	Nebulized budesonide 2 mg 3 times/ Day for 7 d	Oral prednisone tablets 30 mg once a day for 7 days	Oxygen inhalation, antibiotics and bronchodilator treatment	Change in PaO_2, $PaCO_2$, FEV_1, Dyspnea score (Borg score) at 24 h, 72 h, and 7 d control

Supplementary Table 3. Characteristics of the included Observational Studies

Study (year)	Study design	Duration of treatment	Composition of patients	No. of patients (NCs/SCs)	Sex (M:F)		Age (year)		Treatment comparisons		Additional treatments	Outcomes of interest	NOS SCORE
					NCs	SCs	NCs	SCs	NCs	SCs			
Chen et al (2020)	Cohort study	Until discharge	Inpatient	1800 (1091/709)	790:301	564:145	72.9±9.79	72.9±9.59	NR	NR	NR	Length of hospital stay, mortality during hospitalization, and change in arterial blood gases (PaO_2, $PaCO_2$, SaO_2, and pH) from baseline	7
Gu et al (2021)	Cohort study	7 d	Inpatient	45(25/21)	19:6	16:5	78.25±3.54	78.39±3.51	Budesonide 0.25-0.5 mg, inhalation treatment, 2-3 times a day	Methylprednisolone intravenous drip therapy, 40mg/d	Oxygen therapy, relieving spasm and relieving asthma, resolving phlegm and relieving cough are combined with azithromycin	Change in FEV_1% pred, PaO_2, $PaCO_2$, improvement effect and treatment efficiency at 7days control and adverse events	8
Jiang et al (2020)	Cohort study	7 d	Inpatient	100(50/50)	27:23	26:24	55.1±12.3	56.0±11.6	Nebulized Budesonide, 2mg/, 15min/, 2 times / d	Methylprednisolone intravenous drip therapy, 40mg/d	Salbutamol combined with aerosol inhalation therapy, 5mg/, 15min/, twice a day	Change in CRP, FEV_1, FEV_1/FVC, improvement effect and treatment efficiency at 7days control	8
Liao et al (2021)	Cohort study	7 d	Inpatient	50(25/25)	14:11	13:12	42.08±10.54	41.32±10.01	Nebulized Budesonide, 2mg/, 2 times / d	Methylprednisolone intravenous drip therapy. 10 mg/kg/d, 3 d, and then reduced to 5 mg/kg/d, 4 d	Expectorant and antiasthmatic, application support, anti-infection and oxygen therapy	Change in CRP, TNF-α, FVC, FEV_1 at 7days control and adverse events	7
Shi et al (2018)	Cohort study	Until discharge	Inpatient	191(96/95)	63:33	61:34	61.23±14.34	60.73±16.34	Nebulized budeso-nide 2 mg, 3 times/ day	Methylprednisolone intravenous drip therapy, 40mg/d	Terbutaline aerosol 5mg atomization inhalation, 3 times a day	Change before treatment, 5 days, 10 days and 15 days after treatment; Changes of FEV_1 % pred and PEF max and adverse events	8
Song et al (2015)	Cohort study	Until discharge	Inpatient	93(65/28)	(1) 26:7<br>(2) 25:7	22:6	69.91±8.68<br>69.94±10.34	70.5±9.39	Budesonide<br>(1) 1 mg,3 times/day<br>(2) 2 mg,3 times/day	Methylprednisolone intravenous drip therapy, 40mg/d	Routine treatment such as oxygen inhalation, anti-infection, relieving asthma, resolving phlegm, etc.	The clinical manifestations, Change in PaO_2, $PaCO_2$, adverse reactions and the hospitalization days and treatment costs	8
Wang et al (2014)	Cohort study	Until discharge	Inpatient	90(48/42)	NR	NR	65.0±10.1	67.1±8.9	Nebulized budeso-nide 2 mg, 3 times/ day	Methylprednisolone intravenous drip therapy, 40mg/d	Anti-infection, expectorant, oxygen inhalation, bronchodilator treatment	Change in Pulmonary function(FEV_1% pred), arterial blood gas analysis (PaO_2, $PaCO_2$)at 72 hours and 7 days after treatment; Dyspnea scale	7

Zhao et al (2016)	Cohort study	10 d	Inpatient	85(35/50)	NR	NR	NR	NR	Nebulized Budesonide, 3mg/, 3 times/d	Methylprednisolone intravenous drip therapy, 40mg/d	Intravenous theophylline injection, aerosol inhalation and oxygen inhalation therapy	Change in PaO_2, FEV_1% pred, FVC, TNF-α and IL-6 at 10days control	7
Zheng et al (2019)	Cohort study	Until discharge	Inpatient	1847 (1443/404)	1407:395	308:96	72.5±9.7	73.2±9.6	NR	NR	NR	Length of hospital stay (days) and change in FEV_1, arterial blood gases (PaO_2, SaO_2, $PaCO_2$), and pH after treatment	8

NR: Not reported

Supplementary Table 4. Subgroup characteristics of the included Studies

Study (year)	Study Type	Age	NC daily dose	SC daily dose (IV)	SC daily dose (Oral)	SC Types	Follow-up time (days)	Number of patients	Sex ratio (Male:Female)
Ding et al (2016)	RCT	> 70years	6mg	50mg	/	IV	7	471	4.26
Gong et al (2013)	RCT	> 60years	3mg	50mg	/	IV	7	53	4.89
Gunen et al (2007)	RCT	> 60years	6mg	40mg	/	IV	7	82	4.86
Kafee et al (2016)	RCT	> 60years	2mg	/	40mg	Oral	10	90	6.5
Maltais et al (2002)	RCT	> 60years	8mg→2mg	/	60mg→40mg	Oral	10	133	4.54
Mirici et al (2003)	RCT	> 60years	8mg	40mg	/	IV	10	40	2.64
Nemagouda et al (2014)	RCT	> 60years	8mg	50mg	40mg	IV Oral	5	130	NR
Sun et al (2014)	RCT	> 60years	6mg	50mg	20mg	IV Oral	7	30	1.31
Xiao et al (2020)	RCT	> 50years	3mg	40mg	0.5-1mg/kg	IV Oral	5	60	1.22
Yilmazel Ucar et al (2014)	RCT	> 60years	6mg	50mg	/	IV	Discharge day	86	5.13
Zhang et al (2011)	RCT	> 60years	6mg	100mg	30mg	IV Oral	7	98	2.38
Zhao et al (2019)	RCT	> 60years	8mg	1mg/kg→0.5mg/kg	40mg	IV Oral	7	150	1.42
Zhou et al (2004)	RCT	> 60years	6mg	/	30mg	Oral	7	40	4.71
Chen et al (2020)	Cohort study	> 70years	NR	NR	NR	IV Oral	12	1800	3.04
Gu et al (2021)	Cohort study	> 70years	0.5-1.5mg	50mg	/	IV	7	45	3.18
Jiang et al (2020)	Cohort study	> 50years	4mg	50mg	/	IV	7	100	1.13
Liao et al (2021)	Cohort study	> 40years	4mg	12.5mg/kg→6.25mg/kg	/	IV	7	50	1.17
Shi et al (2018)	Cohort study	> 60years	6mg	50mg	/	IV	10	191	1.85
Song et al (2015)	Cohort study	> 60years	4.5mg	50mg	/	IV	11	93	3.65
Wang et al (2014)	Cohort study	> 60years	6mg	50mg	/	IV	7	90	NR
Zhao et al (2016)	Cohort study	NR	9mg	50mg	/	IV	10	85	NR
Zheng et al (2019)	Cohort study	> 70years	NR	NR	NR	IV Oral	11	1847	1.7

Dose of NC: Dose of Budesonide; Dose of SC: SCs doses were converted to the corresponding Prednisone dose (20 mg Hydrocortisone = 5mg Prednisone = 5mg Prednisolone = 4mg Methylprednisolone).

Supplementary Table 5. Newcastle-Ottawa Quality Assessment Scale for Cohort Studies

Study	Selection				Comparability	Outcome			QUALITY SCORE
	Representativeness of the exposed cohort	Selection of the non exposed cohort	Ascertainment of exposure	Demonstration that outcome of interest was not present at start of study	Comparability of cohorts on the basis of the design or analysis	Assessment of outcome	Was followup long enough for outcomes to occur?	Adequacy of follow up of cohorts	
Chen et al (2020)	☆	☆	☆	☆	☆	☆		☆	7
Gu et al (2021)	☆	☆	☆	☆	☆ ☆	☆		☆	8
Jiang et al (2020)	☆	☆	☆	☆	☆ ☆	☆		☆	8
Liao et al (2021)	☆	☆	☆	☆	☆	☆		☆	7
Shi et al (2018)	☆	☆	☆	☆	☆ ☆	☆		☆	8
Song et al (2015)	☆	☆	☆	☆	☆ ☆	☆		☆	8
Wang et al (2014)	☆	☆	☆	☆	☆	☆		☆	7
Zhao et al (2016)	☆	☆	☆	☆	☆	☆		☆	7
Zheng et al (2019)	☆	☆	☆	☆	☆ ☆	☆		☆	8

Supplementary Table 6. Publication Bias in outcome of interest

Outcome of interest (Study design)	Begg's Test (Pr > |z|)((continuity corrected))	Egger's Test (P > |t|)
FEV_1%pred at 5 to 10d control (RCTs)	1.000	0.535
FEV_1(L) at 5 to 10d control (RCTs)	1.000	0.870
$PaCO_2$ (mmHg) at 7 to 10d control (OSs)	0.221	0.090
$PaCO_2$ (mmHg) at 7 to 10d control (RCTs)	0.266	0.214
PaO_2 (mmHg) at 7 to 10d control (OSs)	0.133	0.060
PaO_2 (mmHg) at 7 to 10d control (RCTs)	1.000	0.348
Hyperglycemia (RCTs)	0.754	0.093
Oropharyngeal symptoms (RCTs)	0.806	0.163
Gastrointestinal symptoms (RCTs)	0.230	0.016

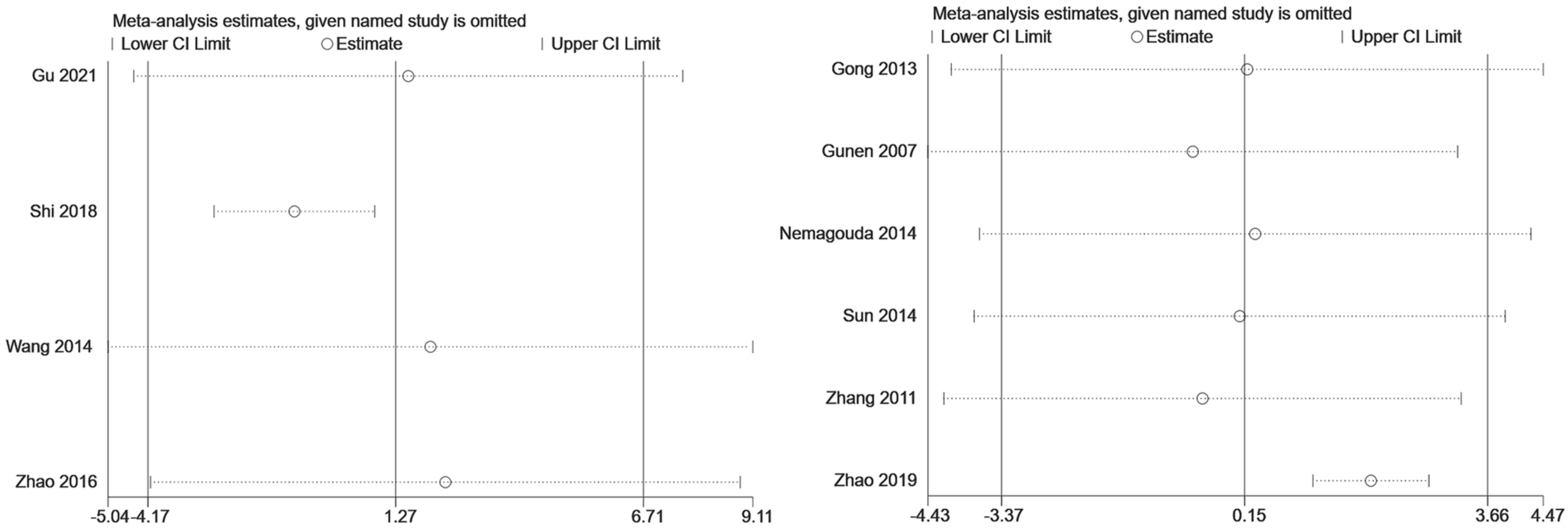

OSs sensitivity analysis RCTs sensitivity analysis

Supplementary Figure 1. Sensitivity analysis of FEV_1% pred at 5 to 10d control in OSs and RCTs

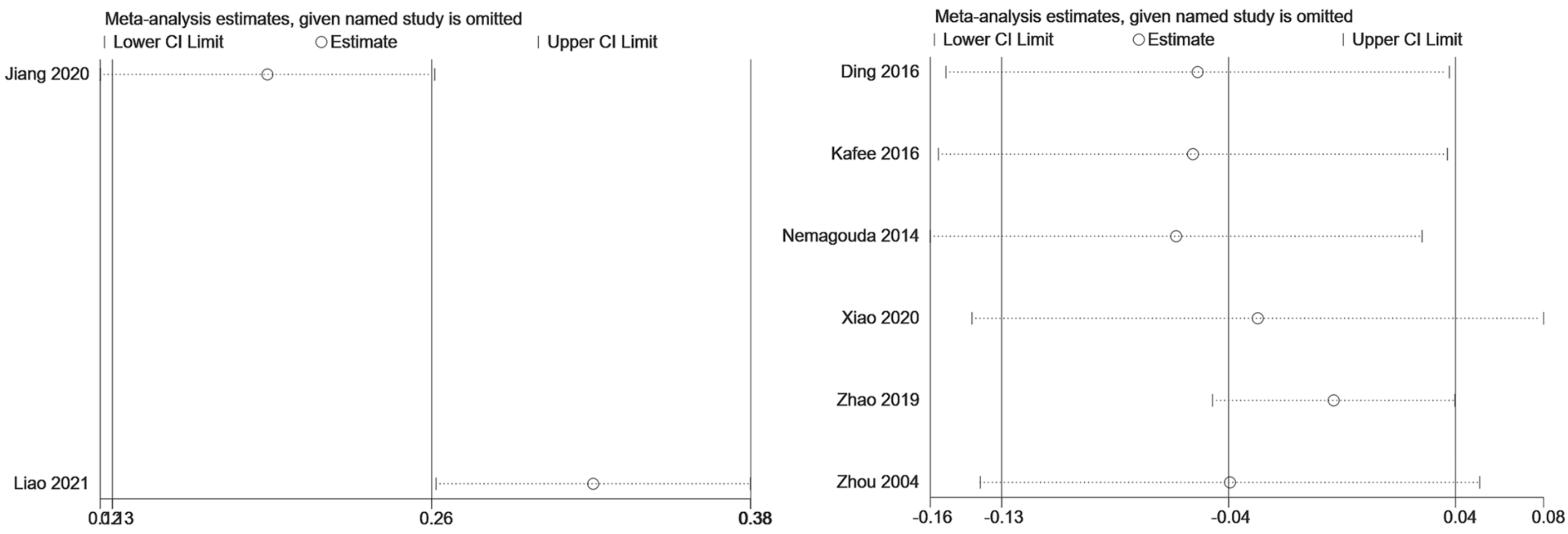

OSs sensitivity analysis RCTs sensitivity analysis

Supplementary Figure 2. Sensitivity analysis of FEV_1(L) at 5 to 10d control in OSs and RCTs

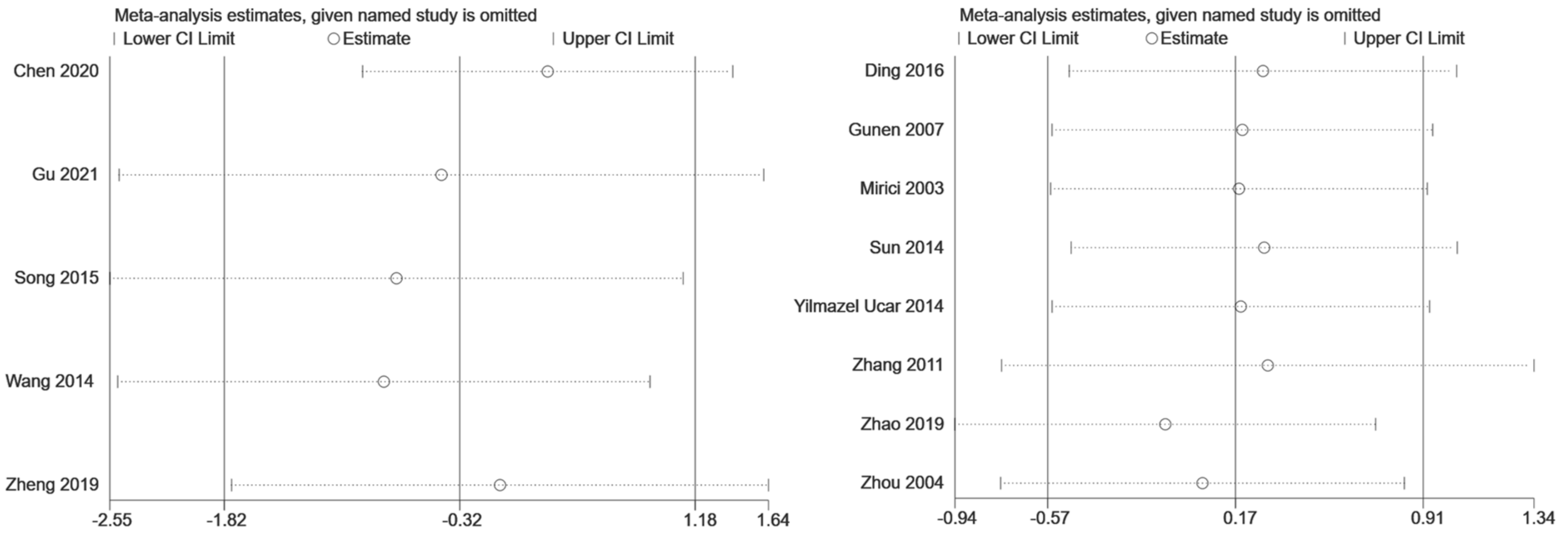

OSs sensitivity analysis　　RCTs sensitivity analysis

Supplementary Figure 3. Sensitivity analysis of $PaCO_2$(mmHg) at 7 to 10d control in OSs and RCTs

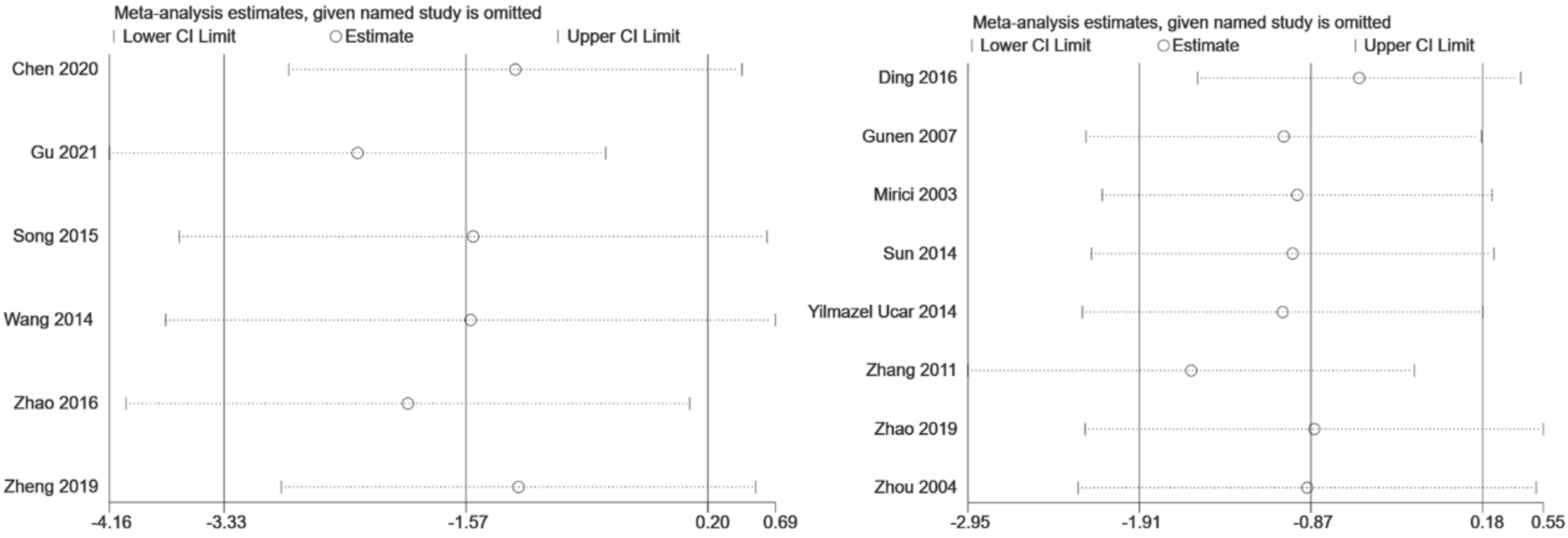

OSs sensitivity analysis　　RCTs sensitivity analysis

Supplementary Figure 4. Sensitivity analysis of PaO_2(mmHg) at 7 to 10d control in OSs and RCTs

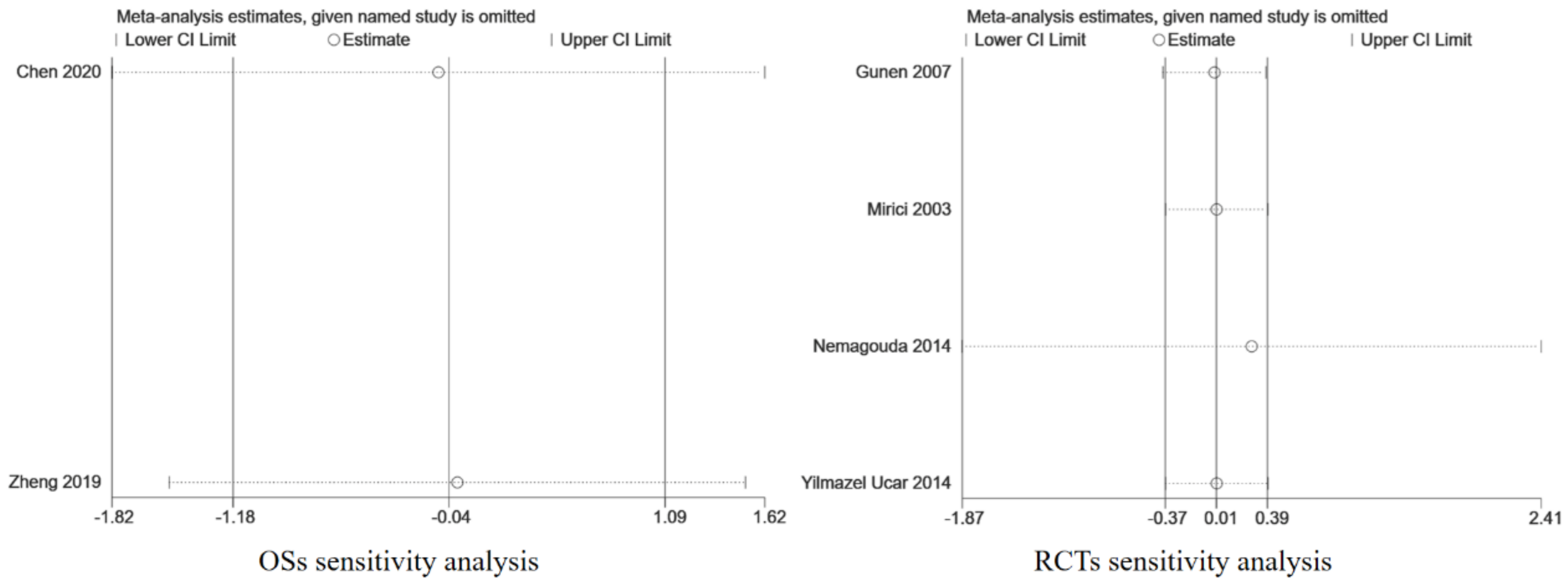

Supplementary Figure 5. Sensitivity analysis of SaO_2(%) at 5 to 10d control in OSs and RCTs

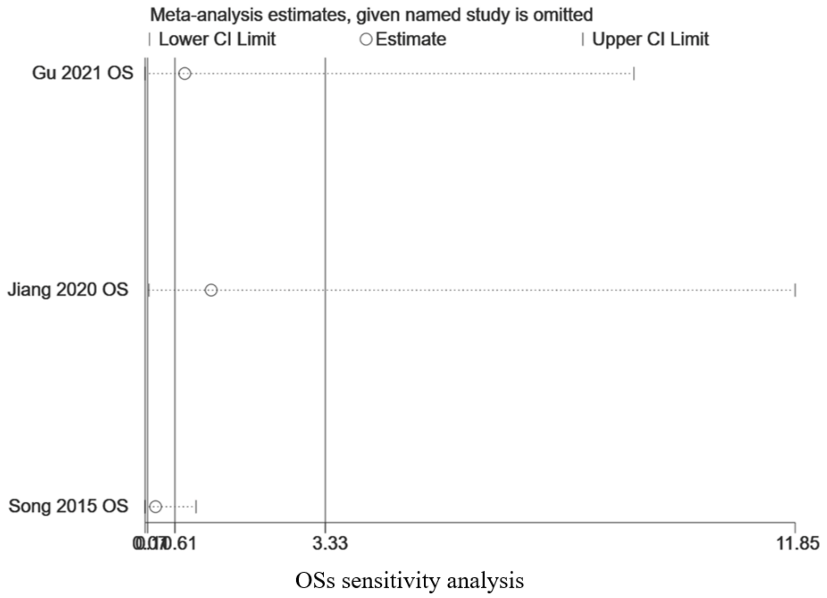

Supplementary Figure 6. Sensitivity analysis of clinical effect in OSs

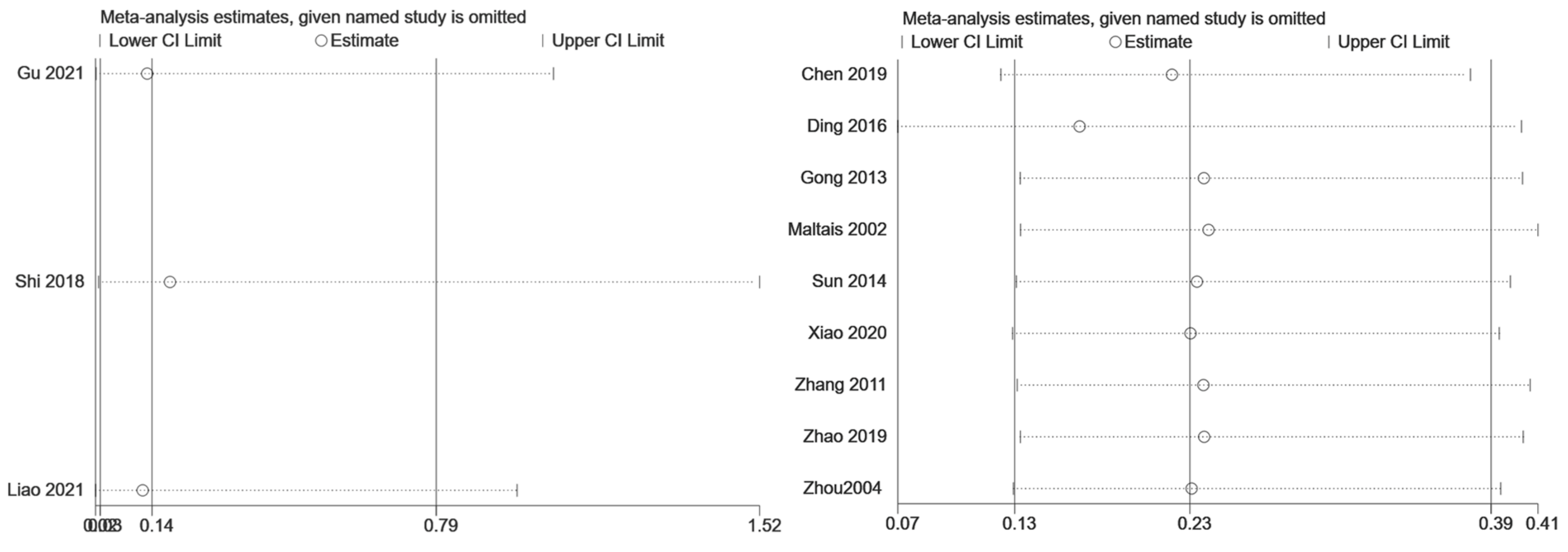

OSs sensitivity analysis RCTs sensitivity analysis

Supplementary Figure 7. Sensitivity analysis of Gastrointestinal symptoms in OSs and RCTs

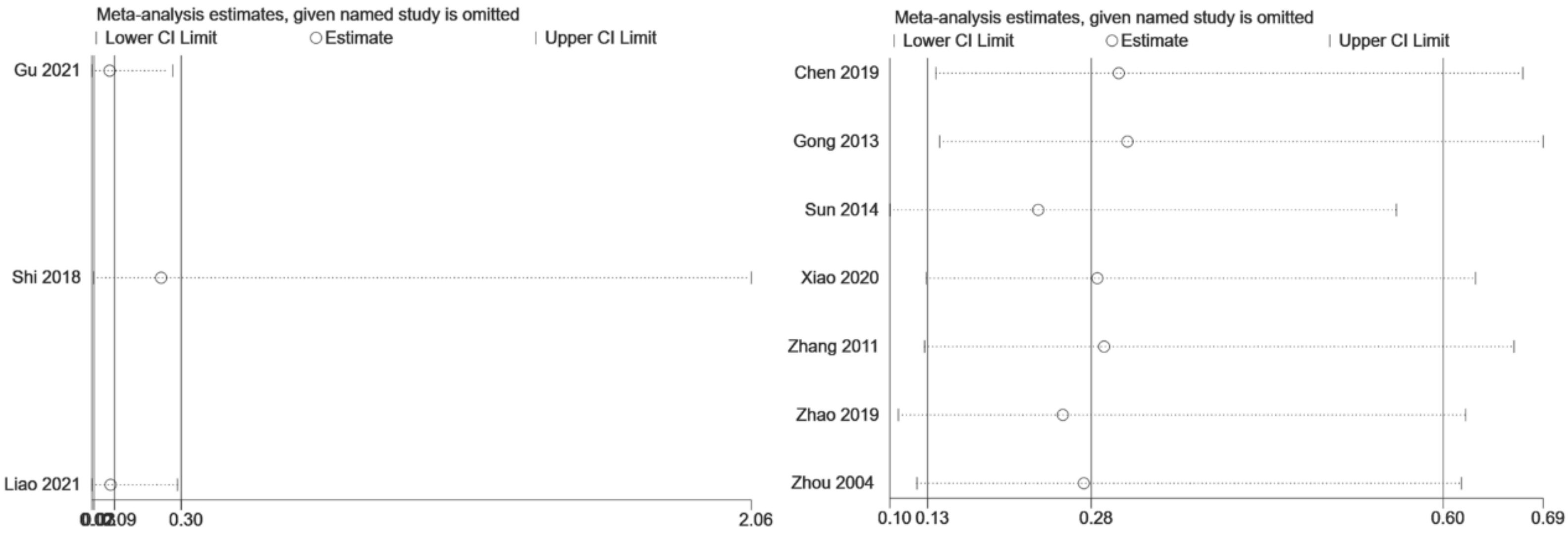

OSs sensitivity analysis RCTs sensitivity analysis

Supplementary Figure 8. Sensitivity analysis of Hyperglycemia in OSs and RCTs

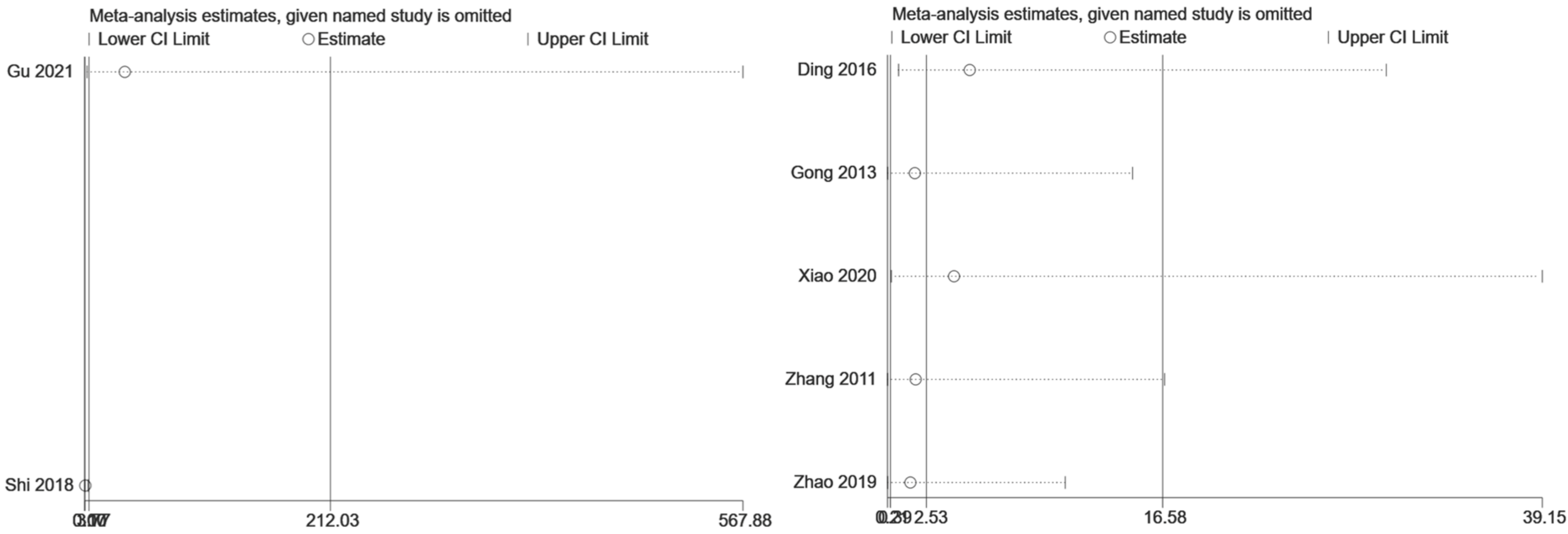

OSs sensitivity analysis

RCTs sensitivity analysis

Supplementary Figure 9. Sensitivity analysis of Oropharyngeal symptoms in OSs and RCTs

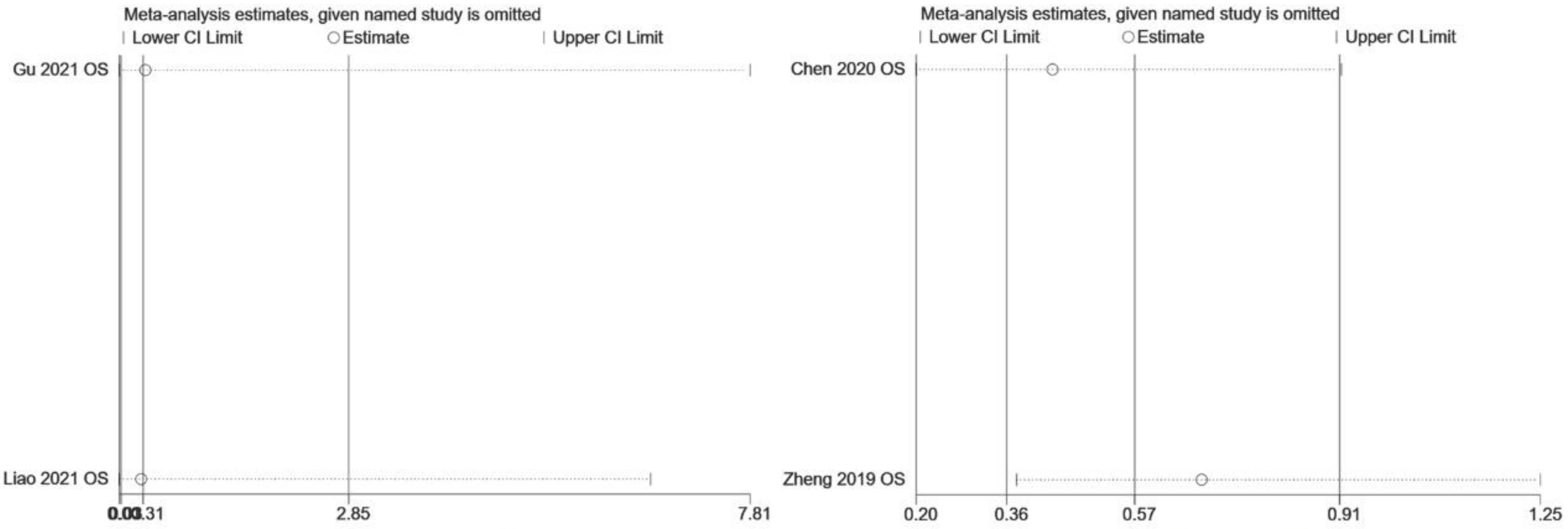

a) OSs sensitivity analysis

b) OSs sensitivity analysis

Supplementary Figure 10. Sensitivity analysis of a) Hypokalemia, b) Pneumonia in OSs

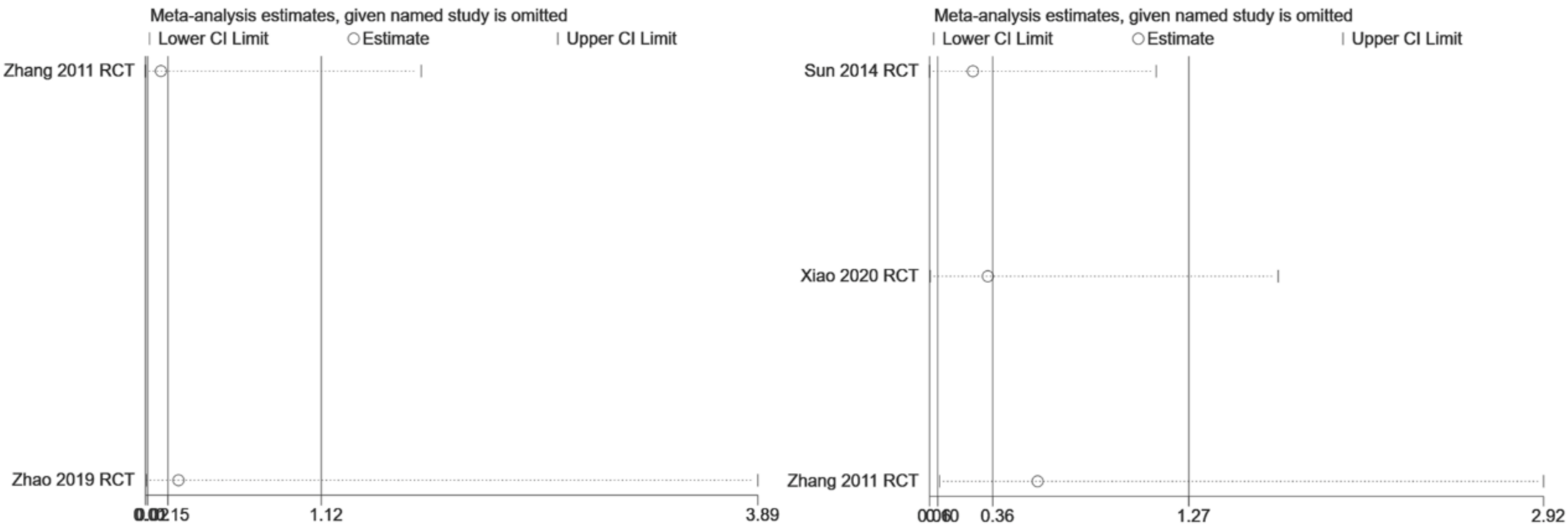

a) RCTs sensitivity analysis b) RCTs sensitivity analysis

Supplementary Figure 11. Sensitivity analysis of a) Elevated blood pressure, b) Excitation and insomnia in RCTs

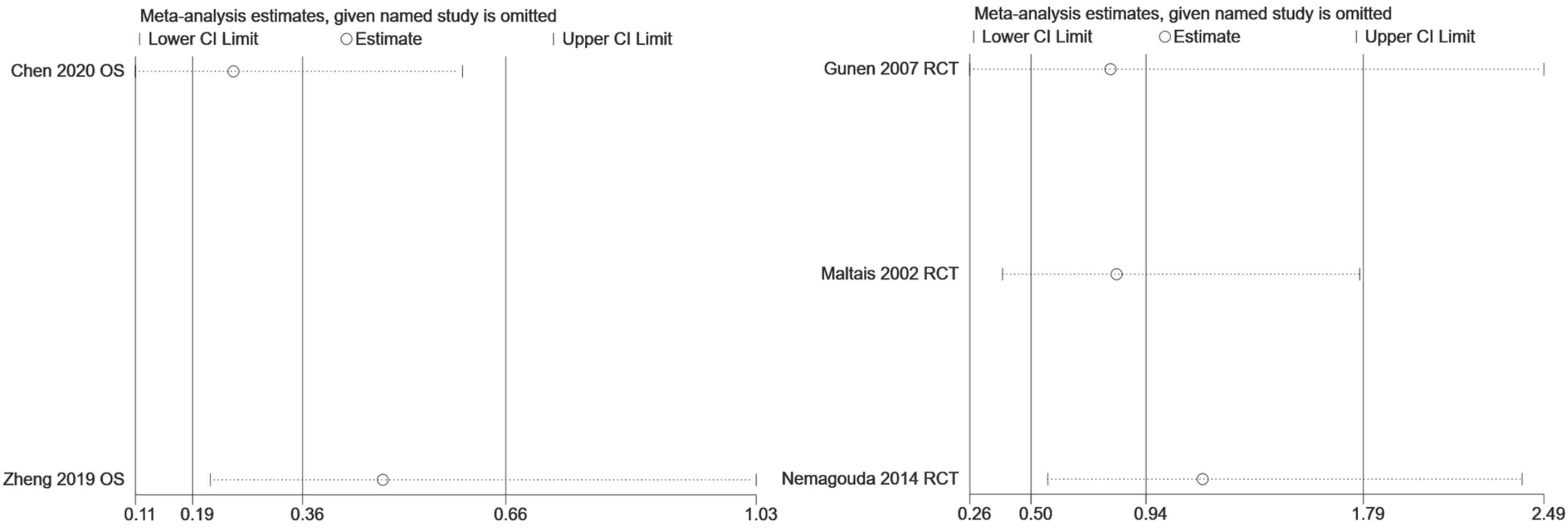

OSs sensitivity analysis RCTs sensitivity analysis

Supplementary Figure 12. Sensitivity analysis of a) Mortality, b) Exacerbations / deterioration in OSs and RCTs

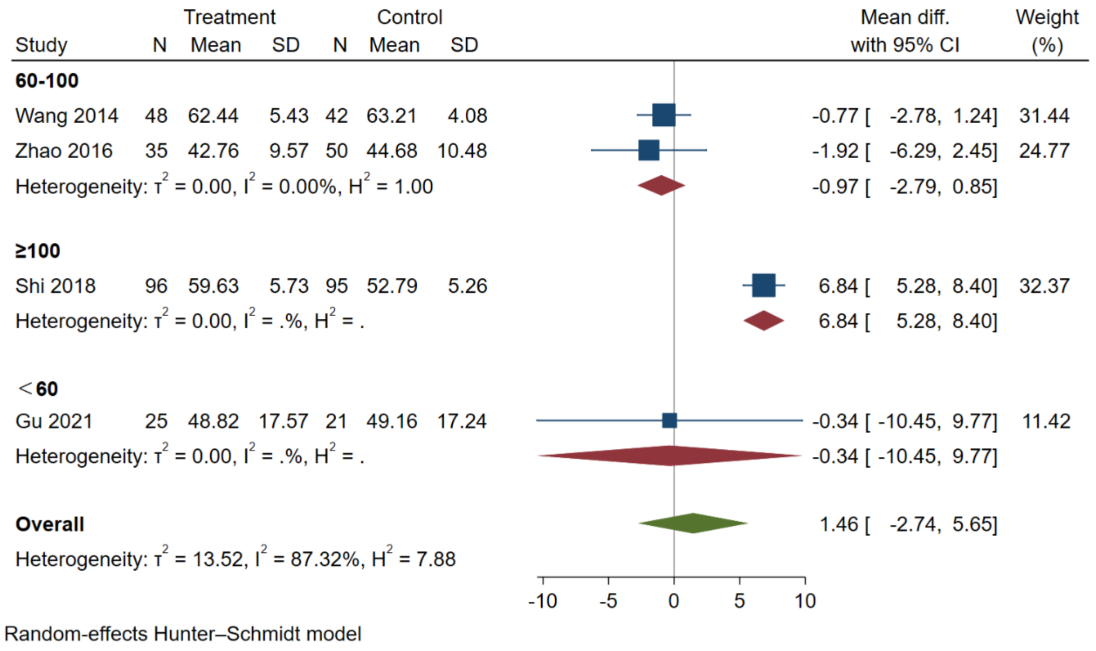

a) Number of patients

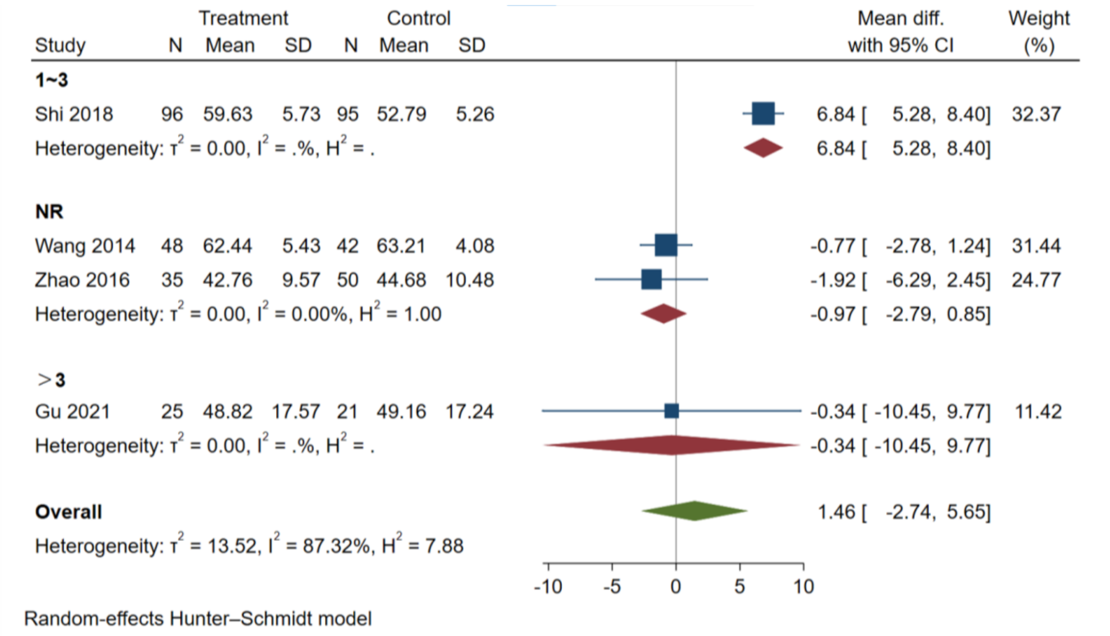

b) Sex ratio (Male number : Female number) (NR: Not Reported)

Supplementary Figure 13. Subgroup (Number of patients, Sex ratio) analysis of FEV_1% pred at 5 to 10d control in OSs

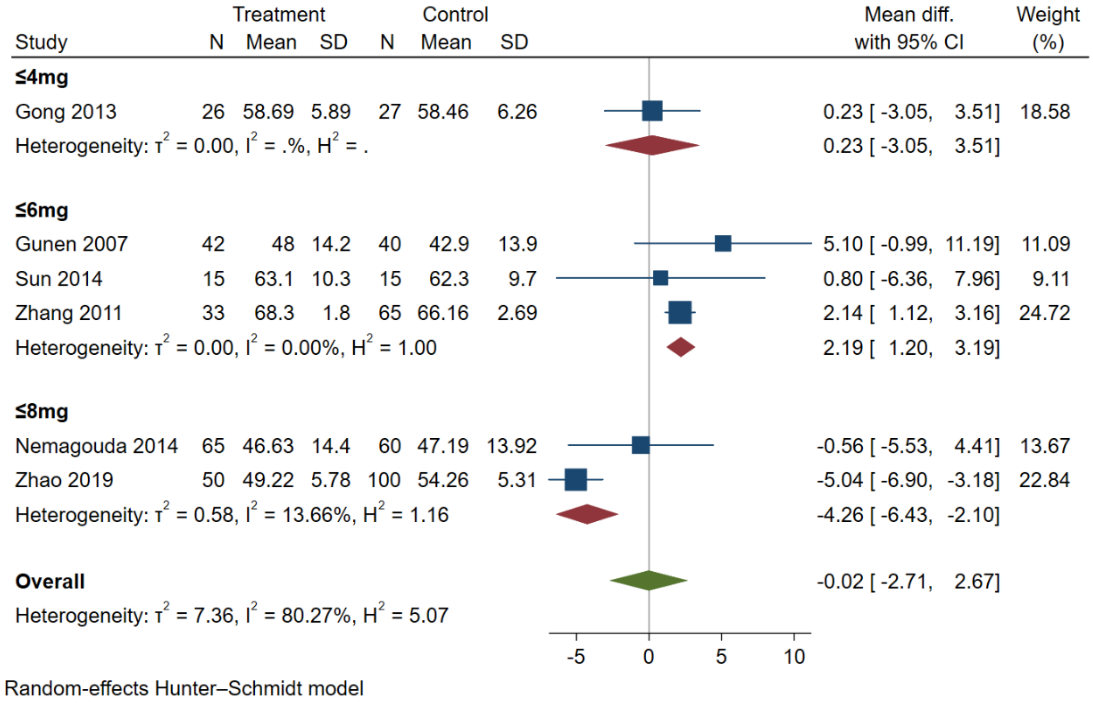

a) Dose of NC

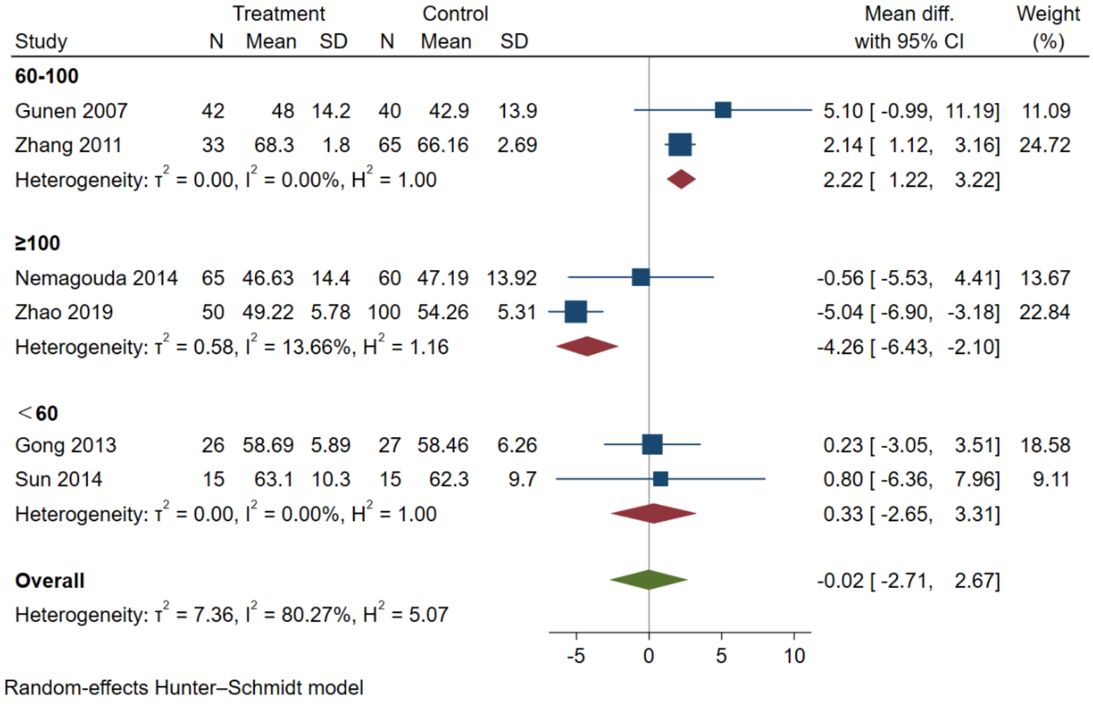

b) Number of patients

Supplementary Figure 14. Subgroup (Dose of NC, Number of patients) analysis of FEV_1% pred at 5 to 10d control in RCTs

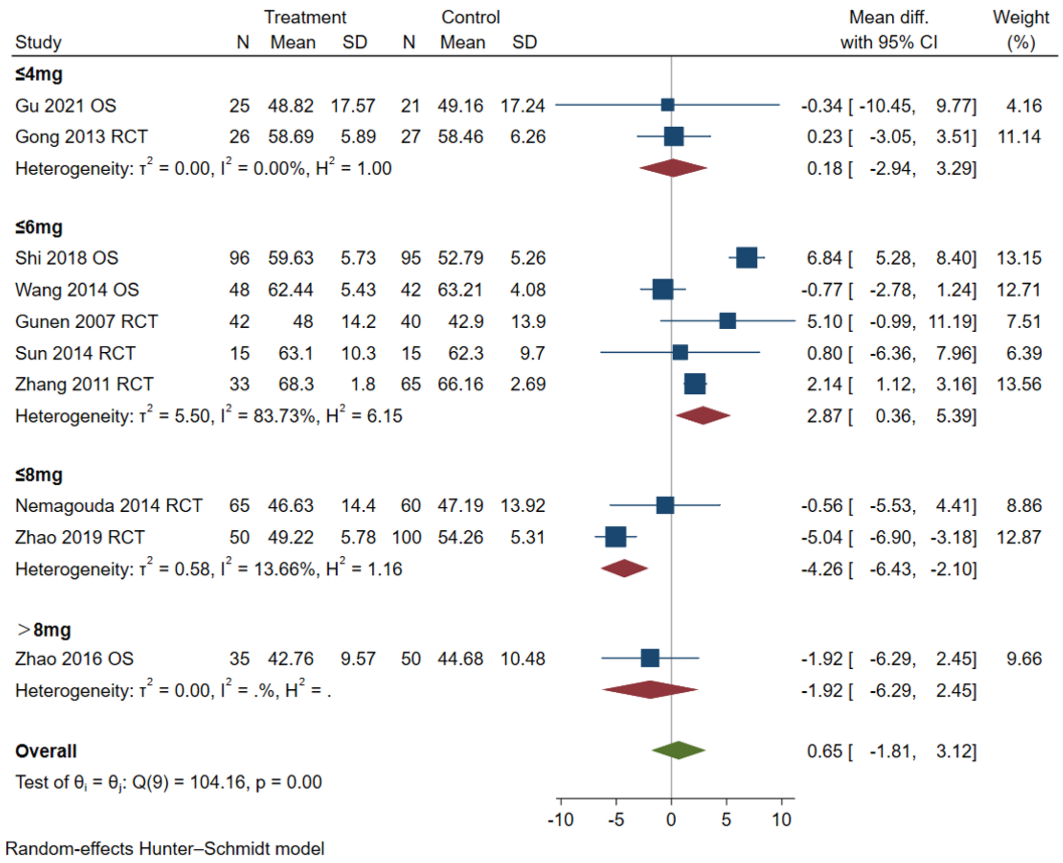

Supplementary Figure 15. Subgroup (Dose of NC) analysis of FEV_1% pred at 5 to 10d control in OSs and RCTs

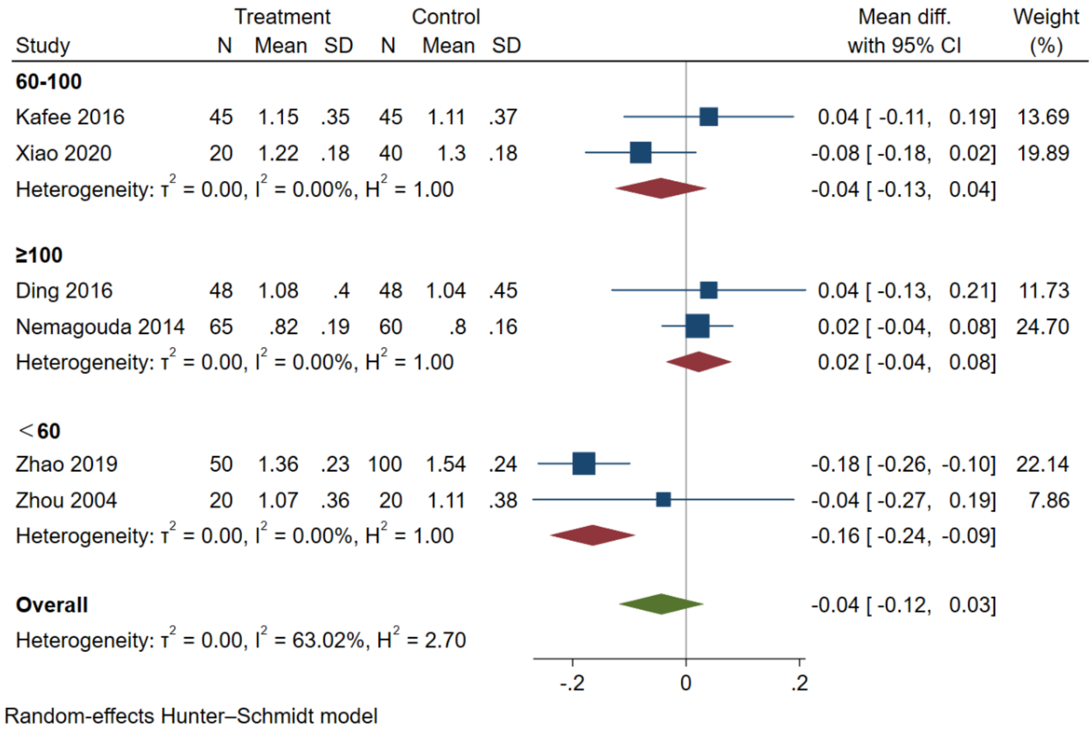

Supplementary Figure 16. Subgroup (Number of patients) analysis of FEV_1(L) at 5 to 10d control in RCTs

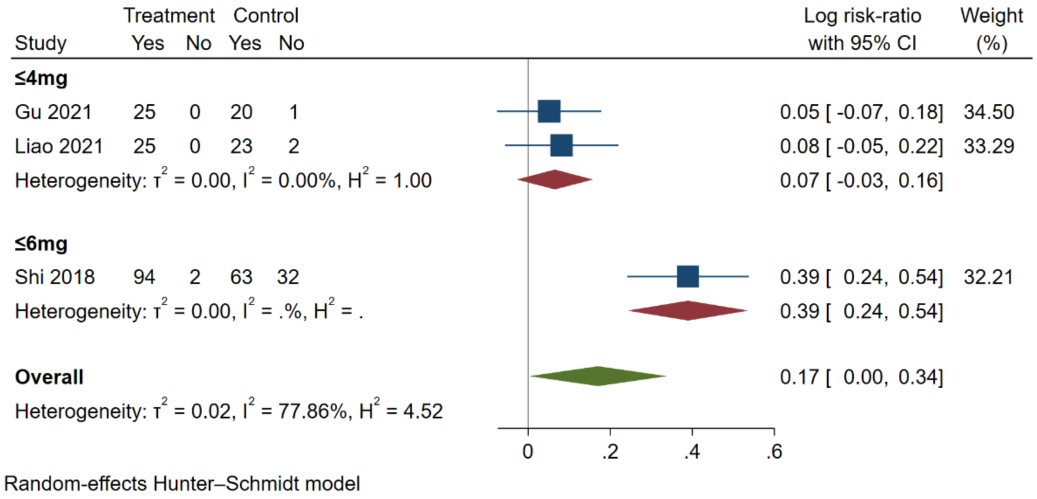

a) Dose of NC

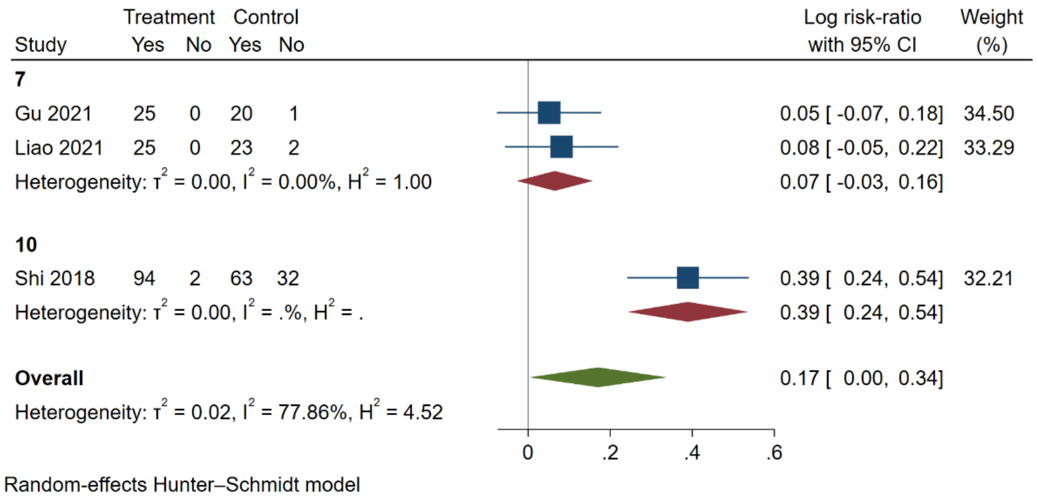

b) Follow-up time

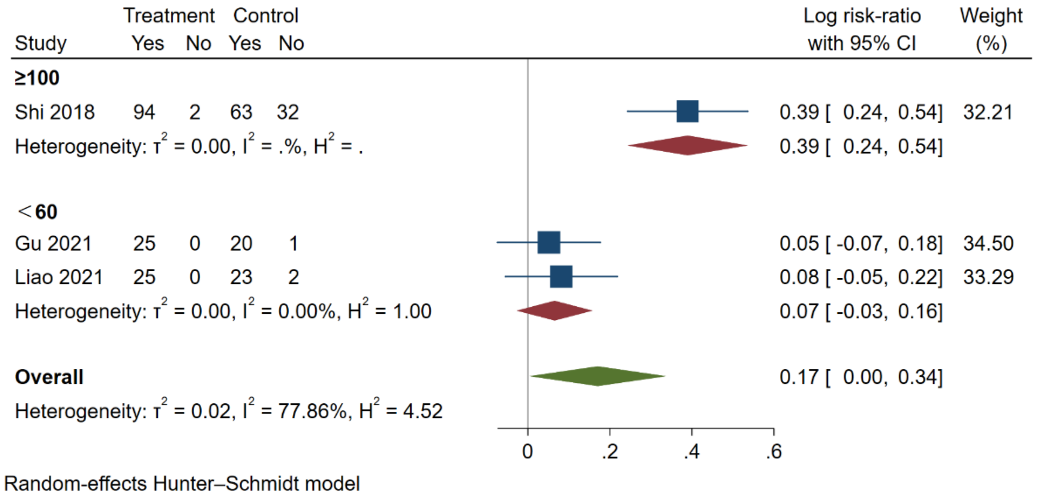

c) Number of patients

Supplementary Figure 17. Subgroup (Dose of NC, Follow-up time, Number of patients) analysis of Gastrointestinal symptoms in OSs

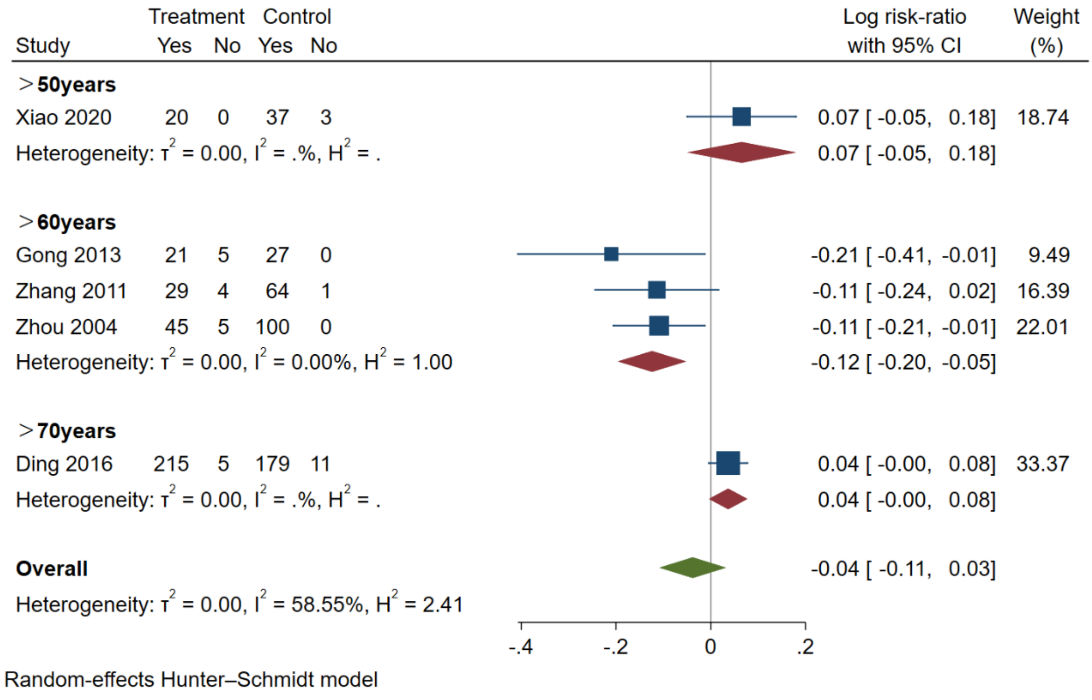

a) Age

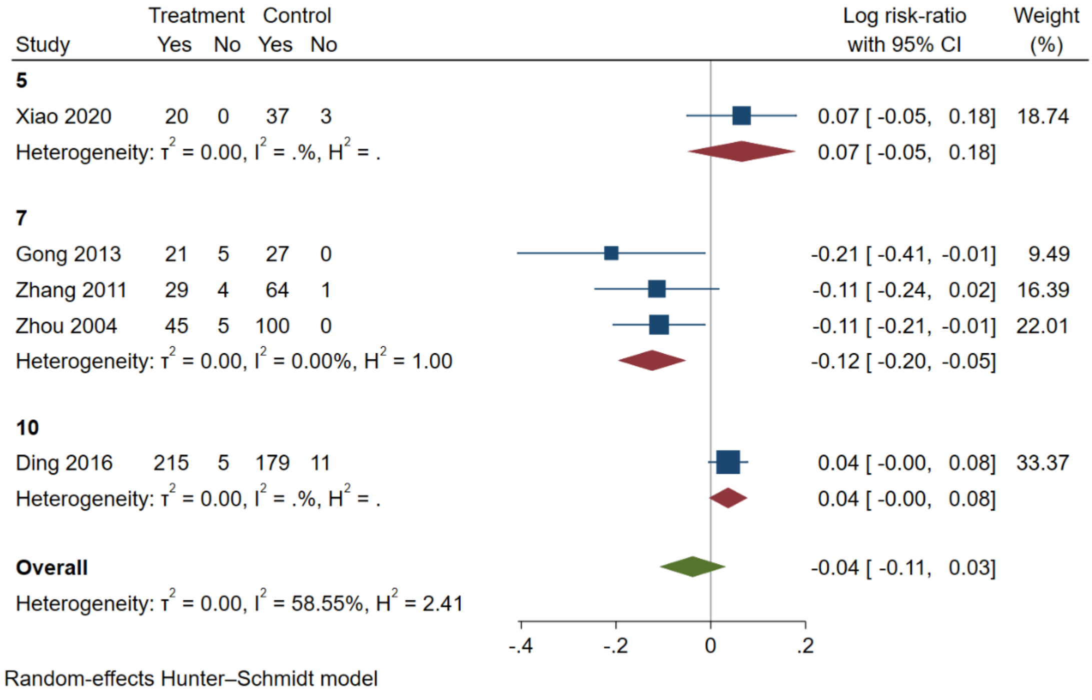

b) Follow-up time

Supplementary Figure 18. Subgroup (Age, Follow-up time) analysis of Oropharyngeal symptoms in RCTs

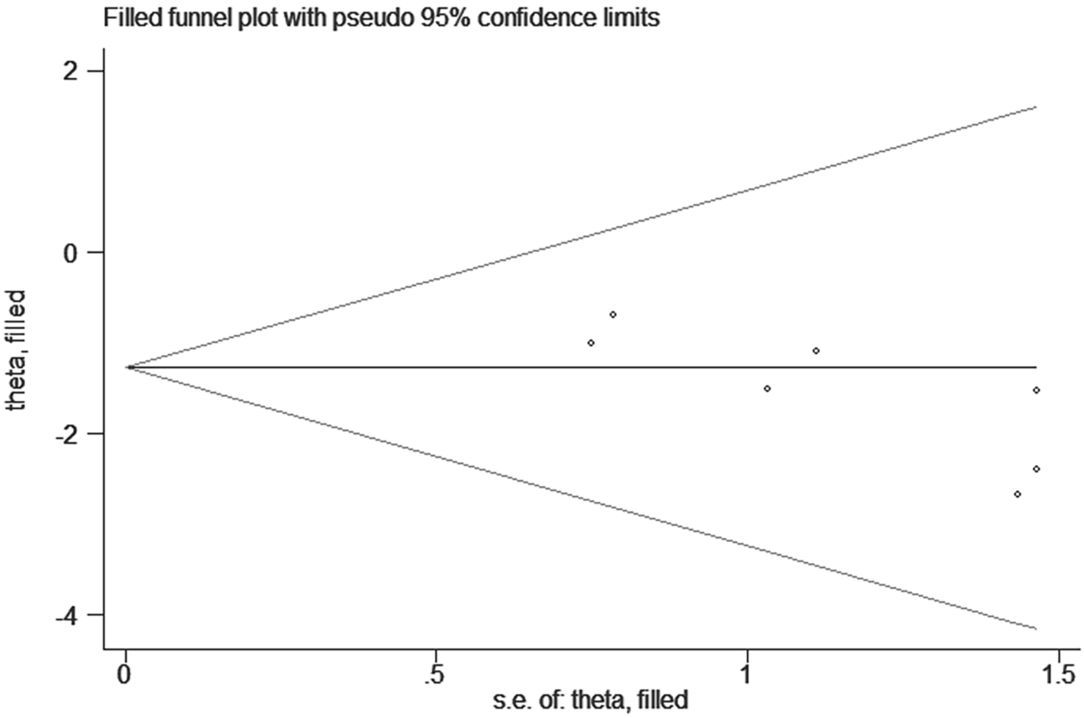

Supplementary Figure 19. Trim and fill method for Gastrointestinal symptoms in RCTs
